# Genome-Wide Analysis of the Pho Regulon in a *pstCA* Mutant of *Citrobacter rodentium*

**DOI:** 10.1371/journal.pone.0050682

**Published:** 2012-11-30

**Authors:** Catherine Cheng, Matthew J. Wakefield, Ji Yang, Marija Tauschek, Roy M. Robins-Browne

**Affiliations:** 1 Department of Microbiology and Immunology, The University of Melbourne, Parkville, Victoria, Australia; 2 Bioinformatics Division, Walter and Eliza Hall Institute of Medical Research, Parkville, Victoria, Australia; 3 Department of Genetics, The University of Melbourne, Parkville, Victoria, Australia; 4 Murdoch Childrens Research Institute, Royal Children’s Hospital, Parkville, Victoria, Australia; University Medical Center Utrecht, The Netherlands

## Abstract

The *p*hosphate-*s*pecific *t*ransport operon, *pstSCAB*-*phoU*, of Gram-negative bacteria is an essential part of the Pho regulon. Its key roles are to encode a high-affinity inorganic phosphate transport system and to prevent activation of PhoB in phosphate-rich environments. In general, mutations in *pstSCAB-phoU* lead to the constitutive expression of the Pho regulon. Previously, we constructed a *pstCA* deletion mutant of *Citrobacter rodentium* and found it to be attenuated for virulence in mice, its natural host. This attenuation was dependent on PhoB or PhoB-regulated gene(s) because a *phoB* mutation restored virulence for mice to the *pstCA* mutant. To investigate how downstream genes may contribute to the virulence of *C. rodentium*, we used microarray analysis to investigate global gene expression of *C. rodentium* strain ICC169 and its isogenic *pstCA* mutant when grown in phosphate-rich medium. Overall 323 genes of the *pstCA* mutant were differentially expressed by at least 1.5-fold compared to the wild-type *C. rodentium*. Of these 145 were up-regulated and 178 were down-regulated. Differentially expressed genes included some involved in phosphate homoeostasis, cellular metabolism and protein metabolism. A large number of genes involved in stress responses and of unknown function were also differentially expressed, as were some virulence-associated genes. Up-regulated virulence-associated genes in the *pstCA* mutant included that for DegP, a serine protease, which appeared to be directly regulated by PhoB. Down-regulated genes included those for the production of the urease, flagella, NleG8 (a type III-secreted protein) and the *tad* focus (which encodes type IVb pili in *Yersinia enterocolitica*). Infection studies using C57/BL6 mice showed that DegP and NleG8 play a role in bacterial virulence. Overall, our study provides evidence that Pho is a global regulator of gene expression in *C. rodentium* and indicates the presence of at least two previously unrecognized virulence determinants of *C. rodentium*, namely, DegP and NleG8.

## Background

Inorganic phosphate (P_i_) is essential for bacterial growth but may be scarce in some environments [1]. Accordingly, microorganisms must be able to regulate P_i_ uptake and to adapt and survive in P_i_ -limiting environments. When P_i_ is not readily available, many species of bacteria activate an adaptive response to scavenge and utilize other forms of phosphorous. In *Escherichia coli* and many other bacterial species, the genes that encode this adaptive response belong to a two-component regulatory system (TCR) called the phosphate (Pho) regulon. This TCR comprises PhoR, an inner-membrane histidine kinase sensor protein, and PhoB, a response regulator that is a DNA-binding protein [1], [2]. A key component of the Pho regulon is the Pst system, a high-affinity P_i_ transport system which captures periplasmic P_i_ and transports it into the cytosol especially when P_i_ is limiting. Besides being a P_i_ transporter, the Pst system also regulates the entire Pho regulon by preventing activation of PhoB through PhoR phosphorylation in phosphate-rich environments. Thus, in *E. coli*, mutations in the Pst system lead to constitutive expression of the Pho regulon regardless of the availability of environmental phosphate. Although PhoB is normally activated by PhoR, it is also subject to cross-regulation by other sensor systems, such as the BaeSR (in *Salmonella* Typhimurium), CreBC (in *E. coli*) and NarQP (in several species of bacteria) TCRs, in response to environmental signals other than P_i_ [3], [4], [5].

The Pho regulon has also been implicated in the virulence of various bacterial pathogens [6]. For example, we have shown that a *pst* mutant of *Citrobacter rodentium* is significantly less virulent for mice, its natural host [7]. *C. rodentium*, a member of the Enterobacteriaceae, belongs to a group of enteric pathogens which induce attaching and effacing (A/E) lesions in intestinal epithelial cells [8]. Other members of this group include enterohemorrhagic and enteropathogenic *E. coli*. All of these A/E pathogens carry a chromosomal pathogenicity island, known as the locus of enterocyte effacement (LEE), which encodes a type III protein secretion system, an outer-membrane protein adhesin (called intimin and encoded by the *eae* gene), a translocated intimin receptor (Tir), and other type III-secreted proteins, most of which are required for the production of A/E lesions [9]. Although inactivation of *pst* affects the virulence of *C. rodentium*, the underlying mechanism of this attenuation is not known. *pstCA* mutants of *C. rodentium* retain the ability to cause A/E lesions, yet they colonize the intestine less efficiently than the wild-type [7].

To understand how inactivation of the Pst system affects the virulence of *C. rodentium*, we used microarray analysis to investigate the global influence of Pst/Pho on gene transcription in *C. rodentium* strain ICC169. These studies allowed us to examine the contribution of Pst and Pho, and the genes they regulate directly or indirectly to the expression of virulence-associated genes by *C. rodentium*.

## Materials and Methods

### Bacterial Strains, Media and Culture Conditions

The bacterial strains and plasmids used in this study are listed in Table 1. Strains were maintained on Luria-Bertani (LB) media and grown overnight at 37°C with shaking unless otherwise stated. Where necessary, antibiotics were used at the following concentrations per milliliter: ampicillin (Amp, 100 µg), kanamycin (Kan, 50 µg), tetracycline (Tet, 12.5 µg), trimethoprim (Tmp, 40 µg), chloramphenicol (Cam, 25 µg), and nalidixic acid (Nal, 50 µg). To detect alkaline phosphatase activity, 5-bromo-4-chloro-3-indolyl-phosphate (XP) was used at a final concentration of 50 µg/ml, together with 0.2% (w/v) glucose. To grow bacteria in known concentrations of phosphate, morpholinepropanesulfonic acid (MOPS) minimal medium [10] containing 0.4% (w/v) glucose, 0.01 mM Casamino acids, and 0.01 mM thiamine was made without added phosphate, after which various amounts of K_2_HPO_4_ were added to a final concentration of 1.0 mM for high phosphate medium (HPM), 0.5 mM for medium phosphate medium or 0.2 mM for low phosphate medium, respectively. Detection of urease activity was performed by using urea-indole medium (Sigma-Aldrich), which was heavily inoculated with bacteria and incubated at 37°C for up to 7 days.

**Table 1:** Bacterial strains and plasmids used in this study.

Strains	Relevant characteristic(s)	Reference or source
***E. coli*** ** strains**		
TOP10	F^–^ *mcrA* Δ*mrr*-*hsdRMS*-*mcrBC*) Ф80*lacZ*ΔM15 Δ*lacX74 nupG recA1 araD139* Δ (*ara*-*leu*)*7697 galE15 galK16 rpsL* (Str^r^) *endA1*λ^–^	Invitrogen
***C. rodentium*** ** strains**		
ICC169	Derivative of *C. rodentium* ICC168, Nal^r^	[97]
ICA15	ICC169 Δ*pstCA::kan* Kan^r^ Nal^r^	[7]
ICA30	ICC169 Δ*degP::kan* Kan^r^ Nal^r^	This study
ICA32	ICC169 Δ*ureB::kan* Kan^r^ Nal^r^	This study
ICA34	ICC169 Δ*nleG8::kan* Kan^r^ Nal^r^	This study
ICA36	ICC169 ΔROD03671*::kan* Kan^r^ Nal^r^	This study
ICA38	ICC169 Δ*tad::kan* Kan^r^ Nal^r^	This study
**Plasmids**		
pCR2.1-TOPO	High-copy-number cloning vector; Amp^r^ Kan^r^	Invitrogen
pACYC184	Medium-copy-number cloning vector; Cam^r^ Tet^r^	New England Biolabs
pMU2385	Single-copy-number transcriptional fusion vector; Tmp^r^	[108]
pMU-*degP*	*degP*-*lacZ* transcriptional fusion	This study
pKD4	FRT-flanked Kan^r^ cassette template	[18]
pAC11	2.0-kb fragment containing wild-type *C. rodentium* ICC169 *degP* cloned intothe BamHI/EcoRV sites of pACYC184; Cam^r^	This study
pAC15	1.5-kb fragment containing wild-type *C. rodentium* ICC169 *nleG8* cloned intothe BamHI/EcoRV sites of pACYC184; Cam^r^	This study

### Recombinant DNA Techniques

Routine DNA manipulations were performed using standard techniques, with the reagents and instructions supplied by the kits’ manufacturers [11], [12]. Genomic and plasmid DNA were isolated by using the CTAB method [11] and the Wizard Plus SV DNA Purification System (Promega), respectively. PCR amplifications were performed using Vent proofreading DNA polymerase (New England Biolabs) or Platinum ***Taq***DNA Polymerase High Fidelity (Invitrogen). A TOPO TA cloning kit (Invitrogen) was routinely used for cloning and sequencing of PCR fragments. Synthetic oligonucleotides for PCR and sequencing (Table S1) were obtained from GeneWorks Pty Ltd.

### RNA Isolation and Labeling

*C. rodentium* strains were grown overnight in medium phosphate medium, then diluted 1∶50 into HPM, and grown at 37°C in a shaking water bath to an optical density of OD_600_ = 0.7. Ten milliliters of this culture were immediately mixed with 20 ml of RNAprotect bacterial reagent (Qiagen) at room temperature for 15 min. Cells were pelleted, and RNA was purified using a FastRNA Pro Blue kit (Qbiogene Inc.). RNA samples were then treated with DNase I using RNase-free DNase (Qiagen) before they were purified further using an RNeasy MiniElute kit (Qiagen). Direct labeling of RNA with Cy5- and Cy3-ULS was performed as described in the Kreatech ULS labeling procedure (Kreatech Diagnostics). The quality, concentration and the extent of labeling were determined with an Agilent 2100 bioanalyzer and an ND-1000 spectrophotometer (NanoDrop Technologies).

### Antisense Microarrays

Custom antisense oligonucleotide microarrays were designed by using the Agilent eArray platform (Agilent Technologies). The arrays contained 4,307 open reading frames (ORFs) representing all gene predictions for *C. rodentium* strain ICC168 (the parent of ICC169) available at the Wellcome Trust Sanger Institute website (http://www.sanger.ac.uk/Projects/C_rodentium/). Each ORF was represented by at least three different oligonucleotides.

### Fragmentation, Microarray Hybridization, Scanning, and Analysis

Fragmentation and hybridization were performed at the Australian Genome Research Facility Ltd. (Melbourne, Australia) as described in the Agilent two-color, microarray-based gene expression analysis manual (version 5.7). Following hybridization, all microarrays were washed as described in the manual and scanned by using an Agilent microarray scanner and Feature Extraction software (Agilent). Normalization and data analysis of the raw data were performed by using the limma package in Bioconductor [13], [14]. Raw data have been deposited in microarray database GEO (Gene Expression Omnibus, reference number GSE40906). Two independent assays were performed in duplicate, and genes were considered differentially expressed if they showed an average change of ≥1.5-fold with an adjusted *P* value of ≤0.05. Functional classification was done in accordance with TIGR’s Comprehensive Microbial Resouce [15].

### *In silico* Search for Pho Box(es)

The *C. rodentium* genome was searched for putative Pho binding sites by using RegulonDB [16] and the *E. coli* K12 PhoBR consensus sequence pattern. Information about gene functions and operon organization was obtained from EcoCyc (http://www.ecocyc.org/), KEGG Pathway database (http://www.genome.jp/kegg/pathway.html), RegulonDB, and TIGR’s Comprehensive Microbial Resource (CMR, http://cmr.jcvi.org/tigr-scripts/CMR/CmrHomePage.cgi).

### Real Time Quantitative Reverse Transcriptase-PCR (RT-qRT-PCR)

RT-qRT-PCR was performed with an MxPro-Mx3005P multiplex quantitative PCR system (Agilent Technologies). First-strand cDNA synthesis was performed with 5 µg of total RNA, SuperScript II RT (Invitrogen), and random primers (Invitrogen) according to the manufacturer's recommendations. For the real-time PCR, each 25-µl reaction mixture contained 10 ng cDNA, 300 nM of each specific primer (Table S1), and 12.5 µl 2x SYBR green master mix (Applied Biosystems). A two-step PCR cycling program, with a dissociation analysis program setting was used. It comprised a 15 min, 95°C hot start period followed by 40 cycles of 95°C for 10 sec and 60°C for 30 sec, followed by one period at 95°C for 1 min, 55°C for 30 sec, and 95°C for 30 sec. A dissociation curve analysis was included to detect secondary or nonspecific product formation. All RT-qRT-PCR data were normalized against the housekeeping gene *rrsB*, and the relative expression ratio of the target gene was calculated as described by Pfaffl [17].

### Construction of Nonpolar Mutants

Knockout mutations were constructed in *C.rodentium* by using the λ Red recombination system [18]. First, approximately 0.5 kb of DNA flanking the target genes was amplified by using primer pairs: htrAF/htrAkanR and htrAkanF/htrAR (for *degP*), ureBF/ureBkanR and ureBkanF/ureBR (for *ureB*), ROD03671F/ROD03671kanR and ROD03671kanF/ROD03671R (for ROD03671), nleG8F/nleG8kanR and nleG8kanF/nleG8R (for *nleG8*) and rcpAF/rcpAkanR and rcpAkanF/rcpAR (for *tad*). The Kan resistance gene was amplified from the plasmid, pKD4, by using primers pKD4F and pKD4R. This product, together with each pair of amplified flanking regions, was used as a template in a PCR with primer pairs: htrAF/R (*degP*), ureBF/R (*ureB*), ROD03671F/R (ROD3671), nleG8F/R (*nleG8*) and rcpAF/R (*tad*) (Table S1). The linear constructs were cloned into the TOPO TA cloning vector pCR2.1-TOPO, introduced into *E. coli* K-12 TOP10 cells. The pCR2.1-TOPO constructs were confirmed by sequencing and then used as templates in a PCR to amplify the linear allelic replacement DNA fragment. PCR products were electroporated into wild-type *C. rodentium* cells carrying the Red system expression plasmid, pKD46, and mutants were selected on LB plates supplemented with kanamycin. All mutations were confirmed by PCR and sequencing.

### Construction of *Trans*-complementing Plasmids

Wild-type *nleG8* and *degP* were amplified from *C. rodentium* ICC169 genomic DNA by using primer pairs nleG8cF/cR and htrAcF/cR, respectively. The resultant PCR products were purified and cloned into TOPO TA cloning vector pCR2.1-TOPO, and sequenced. The fragments were then excised from the TOPO TA derivatives by digestion with BamHI and EcoRV, and ligated to BamHI- and EcoRV-digested pACYC184, behind the Tet^r^ promoter.

### Construction of a *degP*-*lacZ* Transcriptional Fusion

The *lacZ* transcriptional fusion used in this study was constructed by PCR amplification of a DNA fragment which spanned the regulatory regions of the *degP* gene, by using *C. rodentium* ICC169 chromosomal DNA as the template and the primers htrAforw and htrArev (Table S1). The PCR fragment was cloned into pCR2.1-TOPO and sequenced. It was then excised and cloned into the appropriate sites of the single-copy plasmid pMU2385 to create a *degP*-*lacZ* transcriptional fusion.

### β-galactosidase Assay

β-galactosidase activity was assayed as described by Miller [19], and its specific activity was expressed in Miller units. The data presented are the results of at least three independent assays in which samples were processed in duplicate.

### Susceptibility to Acid *in vitro*

*C. rodentium* strains were grown to stationary phase and diluted 1 in 10^5^ in PBS at pH from 2.0 to 6.0 with or without 3.4 mM urea. After incubation for 2 h, bacteria were serially diluted and enumerated on LB agar. The counts were compared to those in the initial inoculum. Strains showing greater than 10% survival at pH 2.5 were considered acid resistant [20].

### Animal Experiments

Three-week-old male C57BL/6 mice were bred, housed, and maintained in the Department of Microbiology and Immunology animal facility at the University of Melbourne. Animals in this facility are certified free of infection with *C. rodentium* and other common bacteria, viruses, and parasites of laboratory mice. For single-strain infections of mice, each of nine mice per group was inoculated by oral gavage with approximately 5×10^9^ CFU of an overnight culture of a test strain of *C. rodentium* in 200 µl of PBS. Inocula were retrospectively quantified by plating serial dilutions on duplicate LB agar plates. Control animals received 200 µl of sterile PBS. Fecal samples were recovered aseptically for up to 20 days after inoculation, and the number of viable *C. rodentium* cells per gram of stool was determined by plating on selective medium. The limit of detection was 100 CFU/g feces. For mixed-strain infections, five mice were inoculated perorally with approximately 10^9^ CFU of a mutant or a complemented mutant strain together with an approximately equal number of wild-type *C. rodentium* cells in 200 µl of PBS. Mice were killed 7 days after infection, their colons were excised and the contents removed and serially diluted. These were then spread on duplicate LB agar plates containing appropriate antibiotics to allow comparison of the proportion of wild-type *C. rodentium* bacteria to that of mutant or complemented mutant bacteria. The ability of the mutant or complemented mutant to compete with the wild-type strain was expressed as the competitive index (CI), which was the proportion of mutant or complemented mutant to wild-type bacteria recovered from each animal divided by the proportion of the mutant or complemented mutant to wild-type bacteria in the inoculum [7]. Test strains with a CI of less than 0.5 were considered to be attenuated.

To test the effect of urease *in vivo*, two series of experiments were performed, in which mice were examined 30 min or 3 days after inoculation with bacteria [21]. For the 30 min experiment, mice were starved overnight and then inoculated with approximately 5×10^9^ CFU by oral gavage as described above. After 30 min, mice were killed by CO_2_ inhalation, the stomach and small intestine were removed and placed in 2 ml of PBS. Samples were weighed, homogenized with a Polytron homogenizer (Kinematica), diluted in PBS, and the bacteria were enumerated on selective agar. For the three-day experiments, mice were inoculated by gavage as described above. Three days later, they were killed by CO_2_ inhalation, and the colon and cecum were removed aseptically and placed in 5 ml of PBS. Samples were weighed and homogenized, and bacteria were enumerated as described above. All experimental procedures were approved by the University of Melbourne Animal Experimentation and Ethics Committee and performed in accordance with the guidelines for animal experimentation of the Australian National Health and Medical Research Council.

### Statistical Analyses

Statistical analyses of data other than microarray data were performed by using the Instat and Prism software packages (GraphPad Software). A two-tailed *P* value of <0.05 was taken to indicate statistical significance.

## Results and Discussion

### Comparison of Transcriptome Profiles between Wild-type and *pstCA* Mutant Strains

To determine the effects of a *pst* mutation and the resultant activation of the Pho regulon in *C. rodentium*, we compared the transcriptome of *C. rodentium* ICC169 and its isogenic *pstCA* deletion mutant (ICA15) when grown in HPM. Preliminary studies showed that there was no difference between the growth profiles of the two strains grown in HPM (data not shown) indicating that the *pstCA* mutation did not affect growth under high phosphate conditions.

**Figure 1:**
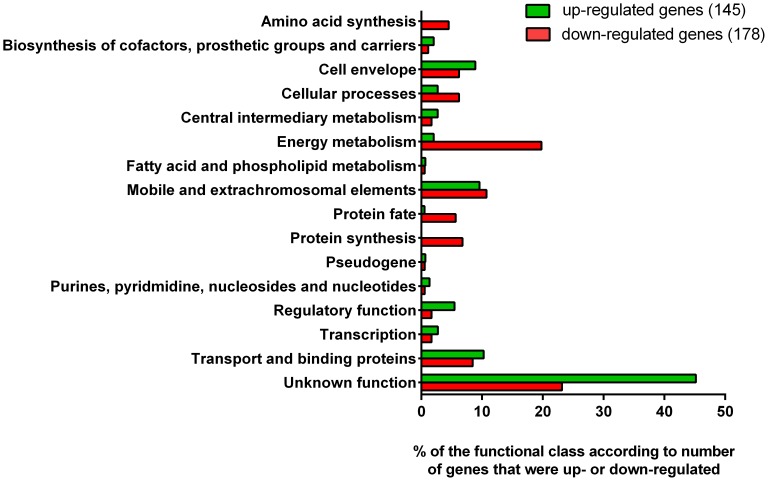
Functional classification of differentially expressed genes in a *pstCA* mutant of *C. rodentium* grown in high phosphate medium. The y-axis shows the functional classification of the genes grouped according to the TIGR’s Comprehensive Microbial Resource, while the x-axis represents the percentage of the functional class according to number of genes that were differentially regulated.

We identified 323 genes that were differentially expressed in the *pstCA* mutant compared to the wild type. Among these genes, 145 (44.9%) were up-regulated and 178 (55.1%) were down-regulated (Tables S2 and S3, respectively). Genes that were expressed differentially by more than 1.5-fold were grouped in functional categories according to the TIGR’s Comprehensive Microbial Resource (CMR) (Fig. 1). The complete list of genes/ORFs that were differentially expressed is provided in Table S4. Among the differentially expressed genes were known Pho regulon genes that encode proteins involved in phosphate metabolism and transport, as well as the phosphate starvation response. Other genes identified were involved in cellular metabolism, transport and binding, and protein fate. Some virulence-associated genes also showed significant transcriptional changes in the *pstCA* mutant. A large number of differentially expressed genes have no known function. To validate the results of the whole-genome microarray, we performed RT-qRT-PCR experiments on 12 representative genes belonging to each functional category (Table 2). The results of these experiments correlated closely with those obtained with the microarray (r = 0.99; *P*<0.0001), confirming that the microarray data reflected the changes in the transcript levels we observed.

**Table 2:** Validation of microarray data by using Real Time quantitative reverse transcriptase PCR analysis of representative genes from each functional category.

Gene/ORF	Functional class	Function	Microarrayfold change^a^	RT-qRT-PCRfold change
*phoE*	Transport and binding protein	outer membrane phosphoporin protein	7.75	6.48
*ytfK*	Unknown function	hypothetical protein	4.91	4.78
*phoA*	Central intermediary metabolism	alkaline phosphatase	3.42	2.90
*katE*	Cellular processes	catalase HPII	2.79	2.15
*mglC*	Transport and binding protein	galactoside ABC transporter	1.13	0.94
*lolA*	Protein fate	outer membrane lipoproteincarrier protein	1.13	0.62
*aidB*	Fatty acid and phospholipid metabolism	isovaleryl CoA dehydrogenase	−1.00	−0.90
*ybdJ*	Unknown function	hypothetical protein	−1.12	−1.01
*ybgA*	Unknown function	putative pathogenicity island protein	−1.57	−1.44
*rplV*	Protein synthesis	50S ribosomal protein L22	−1.74	−1.67
*sucB*	Energy metabolism	dihydrolipoamide succinyltransferase	−2.04	−1.58
ROD03671	Cell envelope	putative F17-like fimbriae	−2.68	−2.28

a Mean of two assays performed in duplicate.

### Identification of Potential PhoB-regulated Genes

Most regulon genes or operons are preceded by a promoter containing an upstream activation site with a consensus sequence for the transcriptional activation by its cognate transcriptional factor. In *E. coli,* the transcriptional factor PhoB binds to a highly conserved DNA sequence called the Pho box, which usually overlaps the −35 region of PhoB-regulated promoters. Most Pho boxes consist of at least two tandem repeats of a 7-bp consensus sequence, 5′-CTGTCAT-3′, separated by an A/T rich 4-bp linker (Fig. S1) [22]. Using computational prediction analysis and the list of genes identified in *E. coli* genomes by Yuan *et al.* [23], a number of predicted Pho boxes were identified among the differentially expressed genes in the *pstCA* mutant. Forty eight possessed putative Pho boxes, including several known Pho regulon members (Tables S2 and S3). These putative genes were found to be distributed across the different functional classes.

### Up-regulated Genes

The majority of the up-regulated genes were classed into four functional groups, namely: (i) hypothetical, unassigned and unknown proteins (*n = *66, 45.2%); (ii) transport and binding proteins (*n = *15, 10.3%); (iii) transcription and regulatory proteins (*n = *12, 8.2%), and (iv) proteins involved in energy and central intermediary metabolism (*n = *6, 4.1%) (Fig. 1; Table S2). Not surprisingly, most of the genes that were strongly induced were either established or predicted Pho regulon genes. Several of these genes are known to be involved in phosphate metabolism, are typically induced by phosphate starvation, and contribute to phosphate transport and uptake. The *ppa* gene, which encodes a cytoplasmic inorganic pyrophosphatase involved in intracellular phosphate metabolism had the highest induction factor overall (11.5-fold). Other established members of the Pho regulon, such as *phoA* for alkaline phophatase, and *psiF* for the metabolism of phosphorous compounds were also significantly induced (3.4- and 4.8-fold, respectively). The genes, *ugpB* for glycerol-3-phosphate and *ugpC* for glycerolphosphoryl diester uptake, were increased 4.8- and 2.8-fold, respectively.

#### Stress response

A large number of genes associated with the RpoS regulon were also up-regulated in the *pstCA* mutant. The RpoS subunit of RNA polymerase is the master regulator of the general stress response in *E. coli*, and is strongly induced when cells are in a nutrient-limiting environment or during the stationary phase of growth [24]. As the Pho regulon is involved in phosphate homeostasis, it is not surprising that the Pho regulon and the RpoS stress response are induced under similar conditions. Previous studies in *E. coli* K-12 have shown that mutations in the *pst* locus, which mimic P_i_ starvation, lead to an accumulation of RpoS [25], [26]. In addition, phosphate-starved *pst* mutants also accumulate the signaling molecule of the stringent response, ppGpp, which promotes transcription of the RpoS-regulatory gene, *iraP* [27]. IraP is an anti-adaptor protein for *rpoS* mRNA stabilization. Its overexpression leads to a significant accumulation of RpoS, and eventually to the expression of RpoS-regulated genes [28]. Therefore, many of the genes up- and down-regulated in the *pstCA* mutant may not be regulated by Pho directly but by RpoS instead.

During exponential growth of the *pstCA* mutant, *rpoS* and *iraP* were up-regulated 1.7- and 1.5-fold, respectively, and 15 genes known to be regulated by the RpoS sigma factor (σ^s^) were significantly differentially expressed. These genes (e.g., *bfr*) are involved in the stress response. They encode metabolic enzymes (e.g., *hycF*), transport proteins (e.g., *artJ* and *ugpBC*) and membrane proteins of unclear function (e.g., *yggE*, *ygiW*, or *yodC*). However, we did not see induction of all known RpoS-dependent genes, suggesting that expression of only a subset of RpoS-regulated genes was induced by changes in phosphate metabolism [29].

#### Survival response

Some genes related to the enhancement of cell survival under harsh conditions were also up-regulated in the *pstCA* mutant. Amongst these were genes induced as part of the oxidative stress response, such as *katE* and *yfiD* (2.8- and 2.5-fold, respectively). KatE catalase protects bacteria by degrading hydrogen peroxide generated as a byproduct of aerobic metabolism. Pyruvate formate-lyase (PFL) catalyzes the conversion of pyruvate to acetyl-coezyme A during glycolysis [30]. The gylcyl radical cofactor of PFL, encoded by the *yfiD* gene, protects bacteria from oxidative stress by facilitating the repair of oxygen-damaged PFL [31]. Gene *yeeD* of the *yeeDEF* operon which was induced 1.6-fold, is also reported to be involved in the oxidative stress response [32]. Genes *puuB* (1.7-fold) and *puuD* (1.6-fold), whose products are involved in the putrescine catabolic process were also upregulated. Putrescine is a polyamine that accumulates in a wide range of organisms, from bacteria to plants and animals, when they experience metabolic stress. It is associated with many functions, including protecting replicating DNA against oxidation [33]. During nutrient starvation, bacteria can make use of the *puuPA-DR-CBE* gene cluster to utilize putrescine as a source of carbon and nitrogen [34]. The up-regulation of genes involved in the survival response indicates that the *pstCA* mutant is subject to environmental stress, which may contribute to its reduced virulence [7], [35].

#### Putative virulence determinants

In addition to up-regulating genes involved in phosphate homeostasis, adaptation and survival responses, inactivation of *pstCA* also activated genes that may increase the virulence of *C. rodentium*. The *dksA* gene (1.7-fold) encodes DksA, a DnaK suppressor protein that regulates the translation of RpoS mRNA. In *Salmonella* Typhimurium, DksA is required for intestinal colonization of newly hatched chickens [36], and in *Vibrio cholerae*, a *dksA* homologue was identified among the genes involved in the colonization of infant mice [37].

*Outer membrane proteins.* Also up-regulated (7.8-fold) in the *pstCA* mutant was the phosphate-starvation-induced pore-forming outer membrane protein, PhoE. In some Gram-negative bacteria (such as *C. rodentium*, *Enterobacter*, *Escherichia* and *Klebsiella* species), the anion-selective PhoE can serve as a conduit for β-lactam antibiotics, chloramphenicol and tetracyclines [38]. Enhanced expression of PhoE can increase the permeability of cell membranes [39], which may contribute to the reduced virulence of *pst* mutants.

In *V. cholerae* O1, a PhoE homologue, encoded by VCA1008, is required for virulence [40]. The incorporation of PhoE in the outer membrane of *V. cholerae* generally leads to a reduction in the amount of outer membrane proteins (OMPs), OmpU and OmpT. In the absence of these porins, PhoE is necessary for *V. cholerae* O1 to infect infant mice. These findings suggest that the *phoE* gene homologue, VCA1008, may play a similar role to that of OmpU under phosphate starvation conditions. OmpU also plays a role in the organic acid tolerance response of *V. cholerae* [41] and is involved in its resistance to bile and other anionic detergents that may be encountered during intestinal colonization [42], [43]. OmpU also contributes to the adhesion of *V. cholerae* to cultured mammalian epithelial cells [44].

*The periplasmic serine protease, DegP, is required for the virulence of C. rodentium.* One particularly interesting result of the microarray analysis was the 2.5-fold up-regulation of the *degP* gene in the *pstCA* mutant. In *E. coli*, DegP is a periplasmic serine protease with proteolytic and general chaperone activities [45], and plays a key role in controlling envelope protein quality [46]. As a protease, it degrades misfolded or aggregated proteins formed after exposure to harmful environments, such as high temperature or oxidative stress. As a chaperone, its primary role is to guide outer membrane proteins through the periplasm. Links between DegP and virulence of *Salmonella* Typhimurium, *Listeria monocytogenes* and *Streptococcus pyogenes* [47], [48], [49], [50] have been demonstrated previously. Although the reason for the reduced virulence of bacteria lacking DegP is uncertain; *degP* mutants may be attenuated because DegP is required to degrade denatured proteins and thus enhance bacterial viability in the face of environmental stresses encountered in the host [51], [52].

In enteropathogenic *E. coli* (EPEC) strain E2348/69, DegP is required for the efficient assembly and expression of the bundle forming pilus (BFP), a type IV pilus and essential virulence determinant that mediates attachment of EPEC to host cells and each other [53], [54], [55]. A *degP_E2348/69_* mutant expresses BFP more slowly than the wild-type strain, a phenotype that can be attributed to the loss of DegP chaperone activity [53]. On the other hand, overexpressing DegP in the periplasm also prevents BFP expression by EPEC, possibly by inducing the degradation of bundlin, the major structural subunit of BFP.

Up-regulation of *degP* in the *pstCA* mutant of *C. rodentium* would lead to overproduction of DegP, which may degrade extracytoplasmic proteins such as bacterial surface adhesins. To explore this further, we constructed a *degP_CR_* deletion mutant, ICA30, and two DegP-overexpressing strains based on the wild-type strain of *C. rodentium* and its *degP* deletion mutant, each of which carried a multicopy plasmid, pAC11,encoding DegP. All three of these strains were then used to infect mice. However, as DegP is involved in maintaining cell envelope integrity and hence, bacterial viability [46], we first determined if the *Citrobacter* derivatives were growth impaired by comparing their growth kinetics with those of the *C. rodentium* parent under various conditions, including growth at 37°C, in Luria broth and in MOPS minimal medium with low and high phosphate concentrations. In every case, ICC169(pAC11) and ICA30 grew at the same rate as each other and the parent strain (data not shown).

**Figure 2:**
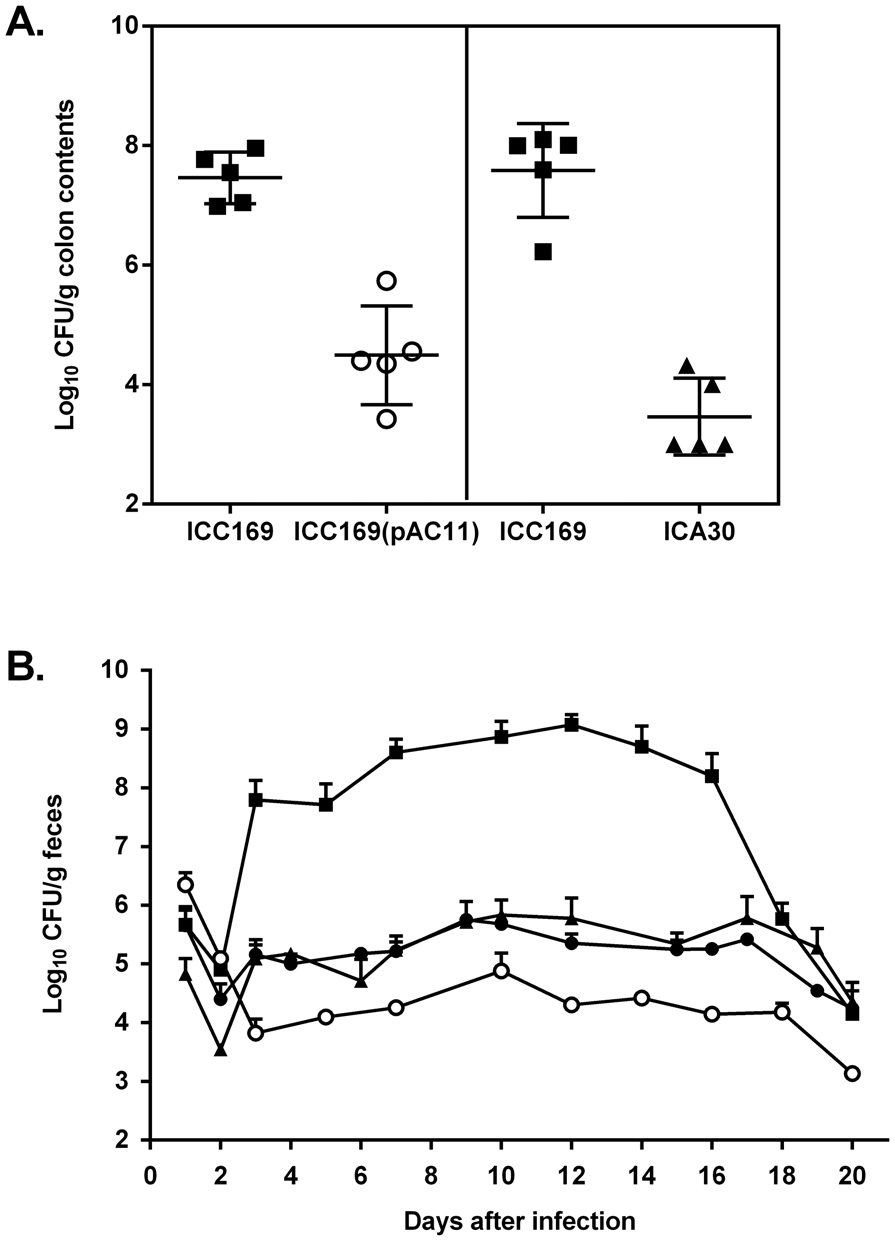
Effect of *degP* on the ability of *C. rodentium* to colonize mice. (**A**) In a competitive infection model, C57/BL6 mice were inoculated by oral gavage with approximately 10^9^ CFU wild-type *C. rodentium* (▪) and either the DegP-overexpressing strain, ICC169(pAC11) (○), or the *degP* mutant, ICA30 (▴), in a 1∶1 ratio. Results are the mean ± SD of the numbers of the two strains in the colon contents 7 days after inoculation. (**B**) C57/BL6 mice were inoculated by oral gavage with approximately 3.6×10^9^ CFU of wild-type *C. rodentium* (▪),or a *degP* mutant, ICA30 (▴), or one of two *degP* overexpressing strains, ICC169(pAC11) (○) or ICA30(pAC11) (•). Results are the mean ± standard deviation log_10_ CFU/g feces from at least four mice. The limit of detection in both experiments was 100 CFU/g feces.

In mixed-strain infection experiments, three-week-old C57/BL6 mice were infected with wild-type *C. rodentium* and either the DegP-overexpressing strain, ICC169(pAC11), or the *degP_CR_* mutant in a 1∶1 ratio. Seven days later, the mice were killed and the ability of the two *C. rodentium* mutant strains to compete with the wild type *in vivo* was assessed by enumerating the test bacteria in the colon. The results showed that both ICC169(pAC11) and ICA30 were out-competed by the wild type with competitive indices (CI) of 0.05 and 0.005, respectively (Fig. 2A). In single-strain infections mice received approximately 3.6×10^9^ CFU of wild-type strain ICC169, ICC169(pAC11), ICA30 or ICA30(pAC11) by oral gavage. The ability of ICC169(pAC11), mutant ICA30, and ICA30(pAC11), to colonize mouse intestine was significantly less than that of the wild type, as shown by the peak mean counts of 7.6×10^4^ CFU/g feces, 6.8×10^5^ CFU/g feces, 5.6×10^5^ CFU/g feces, and 1.2×10^9^ CFU/g feces for strains ICC169(pAC11), ICA30, ICA30(pAC11), and ICC169, respectively (Fig. 2B). These results suggest that C. *rodentium* requires *degP* for virulence, and that both reduced expression and overexpression of DegP reduces the virulence of *C. rodentium*.

#### Other up-regulated genes

Sixty-six hypothetical genes of unknown function, some of which are common to many different species of enterobacteria, also displayed increased expression in the *pstCA* mutant. The *yggE* gene (1.5-fold), conserved in *Shigella flexneri* and *Edwardsiella ictaluri*, encodes a putative extracellular protein. *E. ictaluri* is the causative agent of enteric septicemia of catfish [56], and *yggE* is considered a potential target antigen for vaccines for this disease [57]. Another gene, *ytfK* (4.9-fold), encodes a small protein (68 aa) whose function is not known, but is reported to be up-regulated when *E. coli* forms a biofilm [58]. This observation supports a role for the Pho regulon in biofilm formation [6]. Also up-regulated was the *yibD* gene, a putative glycosyl transferase, which is involved in the metal ion stress response of *E. coli* and is particularly important for resistance to zinc [59], [60]. Several other genes such as *yodC* and *ygiW* also encode putative stress-induced proteins [61].

### Down-regulated Genes

The 146 down-regulated genes in the *C. rodentium pstCA* mutant encode proteins involved in energy and central intermediary metabolism (*n = *39, 21.9%), protein synthesis and fate (*n = *22, 12.4%), cell envelope and cellular processes (*n = *22, 12.4%), or are hypothetical or unknown proteins (*n = *41, 23.2%) (Fig. 1; Table S3). Amongst the down-regulated genes involved in protein fate and synthesis were genes for ribosomal proteins and chaperones involved in protein synthesis, such as *rplACEFJKLMNOQPRSUVWXY* for 30S, *rpsABDEFHJKMNPOQSU* for 50S and *dsbC* (−1.7-fold). *dsbC* encodes a periplasmic protein with protein disulfide isomerase and chaperone activities. Down-regulation of the information processing machinery and chaperones suggests a reduction in cellular processes and a possible increase in unfolded and damaged proteins, which would be deleterious to the cell and may contribute to the stress response and reduced virulence of the *pstCA* mutant. As no putative Pho boxes were identified upstream of these ribosomal genes, however, such changes may not be influenced by the Pho regulon directly.

#### Cellular metabolism

In general, the *pstCA* mutation resulted in decreased expression of genes required for amino acid biosynthesis and energy production. Among the genes involved in amino acid biosynthesis that showed reduced expression were *argE* (−1.7-fold) and *argG* (−1.6-fold) for arginine biosynthesis; *serA* (−2.7-fold), in glycine, serine and threonine biosynthesis, and *aroC* (−1.5-fold) in chorismate biosynthesis. Several studies have shown that when phosphate is limited, genes involved in amino acid biosynthesis are repressed [62], [63], which is consistent with the slower growth rate of cells under P_i_-limitation [64]. As the *pstCA* mutant falsely senses phosphate limitation, it may reduce amino acid synthesis to conserve energy.

Genes involved in energy production and carbohydrate metabolism were also down-regulated in the *pstCA* mutant. These included the membrane-bound ATP synthase (*atpHG,* -1.6 fold), which plays a central role in free-energy transduction under aerobic and anaerobic growth [65]. Expression of *dmsABC*, which encodes dimethyl sulfoxide reductase was also reduced (−1.8-fold). Other down-regulated genes were some for enzymes of the tricarboxylic acid (TCA) cycle, including *gltA* for type II citrate synthase (−1.8-fold), *acnB* for aconitase hydratase 2 (−1.8-fold), *sucAB* (−2.0-fold), and *sucCD* (−1.6-fold). *sucAB* and *sucCD* encode the enzymes, 2-oxoglutarate dehydrogenase and succinyl coenzyme A synthetase, that generate succinyl-CoA from 2-oxoglutarate and succinate, respectively. Besides its central role in the TCA cycle, succinyl-CoA is also used as an intermediate in many metabolic pathways, a major one being the lysine biosynthetic pathway, where succinyl-CoA is used to generate diaminopimelate that is required for the biosynthesis of peptidoglycan [66].

#### Cell membrane components

Several genes involved in the regulation of cell surface components, such as the *proVWX* operon (−1.8-fold) and *ampG* (−1.6-fold), showed reduced expression in the *pstCA* mutant. The *proVWX* operon encodes a high-affinity, binding-protein-dependent transport system for the osmoprotectants, glycine betaine and proline betaine, which play a key role in bacterial survival under osmotic stress [67]. The *ampG* gene encodes a muropeptide permease that is involved in the synthesis and recycling of cell wall peptides [68]. Turnover and recycling of peptidoglycan is a major metabolic pathway of *E. coli*, and decreasing its rate helps the bacteria cope with external stress. Besides its role in peptidoglycan recycling, *ampG* is also required for efficient colonization of the mouse bladder by uropathogenic *E. coli* [69].

Down-regulation of the genes encoding outer membrane porins OmpA (−1.9-fold) and OmpF (−1.7-fold), which are involved in the transport of solutes, indicated that the *pstCA* mutation also affected cell membrane constituents. However, the alternative sigma factor σ^E^ (encoded by *rpoE*), which is activated by envelope stress and promotes expression of factors that help maintain cell envelope integrity [70], [71] was not differentially regulated in the *pstCA* mutant. Hence, the decrease in OmpA and OmpF levels may be due to the periplasmic accumulation of other OMPs, such as PhoE, which may dilute the chaperones and assembly proteins required for the insertion of OmpA and OmpF in the outer membrane. Genes involved in lipopolysaccharide biosynthesis and lipid A modification (e.g., *waaQGP-rfaS-waaBIJY-rfaZ-waaK* and *lpxH*) were also down-regulated, albeit modestly (−1.3-fold), suggesting that these genes are weakly, or not at all, regulated by the Pho regulon in *C. rodentium*. Taken together, the changes in the cell envelope structure that would result from reduced expression of the aforementioned genes may contribute to the reduced virulence of *pst* mutants of *C. rodentium*.

#### Inducible acid survival systems

Enteric bacteria such as *Salmonella enterica* and enterohemorrhagic *E. coli* have evolved a variety of adaptive strategies to manage the broad range of acid stresses encountered in the stomach and some foods. In *E. coli*, the most well studied form of acid resistance is the amino acid-dependent system that enables cells to survive at pH levels less than 3 for several hours. This system is based on the decarboxylation of certain amino acids, such as glutamate, arginine, and lysine, by GadA/B, AdiA, and CadA, respectively, and the importation of these amino acids via their cognate antiporters (GadC, AdiC and CadC, respectively) [72], [73]. Although the importance of this phenotype for the metabolism and environmental fitness of *E. coli* is uncertain, it has been suggested that decarboxylases of this type may counteract acidic conditions and hence serve to control insoluble carbon dioxide and facilitate pH homeostasis. The ability to respond quickly to changes in pH involves the intersection of different regulatory pathways and overlapping control of gene expression. It is not surprising therefore, that low-pH-inducible genes, such as *gadA/BC*, *asr* (acid shock RNA) and *xasA* (acid sensitivity protein), are regulated in part by the Pho regulon [74], [75], [76].

A bioinformatic search showed that *C. rodentium* has lost some of the genes involved in acid stress, in particular, the glutamate-dependent, acid-resistance genes that are required for bacterial survival at pH 2. This may be attributed to a change of niche, where once it shared a common ancestor with *E. coli* and *Salmonella* [8], it now has evolved to become a murine pathogen, and genes required for its previous lifestyle have been lost. Whereas enteropathogens of humans need to tolerate the conditions of a fasting stomach, pH ≤2, *C. rodentium* need only tolerate a pH of ∼3.5, the gastric pH of mice [77]. Hence, an elaborate system of acid resistance may be redundant. Although it does not seem to express glutamate-dependent acid tolerance, *C. rodentium* does encode a full length urease gene cluster (*ureDABCEFG*) and can produce urease. Urease is involved in nucleotide and amino acid metabolism, and converts urea to carbon dioxide and ammonia. Through the generation of ammonia, urease may enhance virulence as it protects bacteria such as *Helicobacter pylori*, *Proteus mirabilis*, *Yersinia enterolitica* and *Brucella abortus* from the deleterious effects of gastric acid [77], [78]. Interestingly, some of the genes belonging to the urease cluster were down-regulated 3.6-fold in the *pstCA* mutant. Although the role of urease in disease has been intensively studied in some bacteria, its contribution to acid resistance and the virulence of *C. rodentium* is not known.

*Urease does not play a key role in acid resistance or virulence of C. rodentium.* To determine if urease contributes to the acid resistance and virulence of *C. rodentium*, we generated a urease *ureB_CR_* deletion mutant, named ICA32. Expression of urease was determined by a qualitative urease assay using urea agar, which turned bright pink within 24 h of incubation with the wild-type strain, but showed no colour change when incubated with either the *pstCA* mutant, ICA15 or the *ureB_CR_* mutant, ICA32, even after 96 h (data not shown).

**Figure 3:**
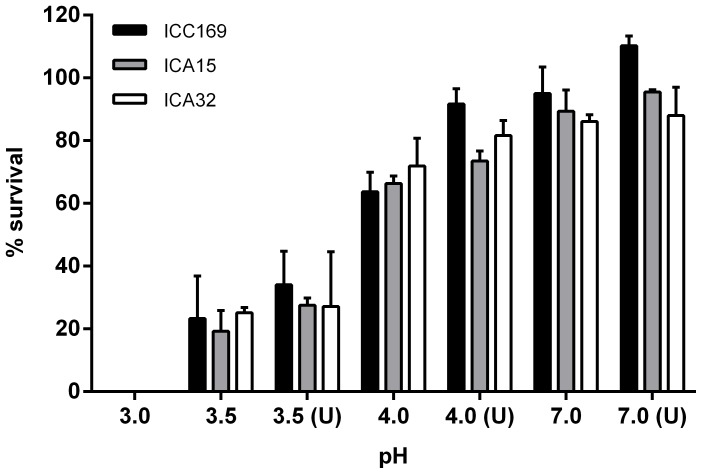
Survival of *C. rodentium* ICC169 and its derivatives in acid. Stationary-phase cultures of *C. rodentium* strains were adjusted to 10^5^ CFU/ml in phosphate buffered saline at various pHs in the absence or presence of 3.4 mM urea (U). After incubation for 2 h at 37°C, surviving bacteria were enumerated on agar, and their numbers were compared to those in the initial inoculum. Data are the mean and standard deviations from three separate experiments.

Since mice have a gastric pH of ∼3.5, *C. rodentium* need only to survive passage through the stomach at pH ∼3.5 for up to 2 h (gastric emptying time) before entering the intestine [77]. To determine the acid resistance of *C. rodentium* ICC169 *in vitro*, we tested its ability to survive in phosphate buffer at pH ranging from 3.0 to 7.0 (Fig. 3). Strains ICA15 and ICA32 were tested concurrently. Wild-type *C. rodentium* was able to survive for 2 h at pH 4.0 and above, but at pH 3.5, its viability was reduced by around 80% and at pH 3.0, all cells were killed. The addition of 3.4 mM urea (a concentration similar to normal serum levels [77]) at pH 3.5 did not significantly increase bacterial survival. The addition of urea at all pH levels tested did enhance growth of the wild type. This may have been due to breakdown of urea to ammonia, a rich source of organic nitrogen, and a growth promoter. Nevertheless, differences in the survival of the wild type, ICA15 and ICA32 were not significant at every pH tested. In all, this indicates that urease may contribute to the growth of *C. rodentium* when the availability of nitrogen is limited, but does not play a key role in acid resistance.

To investigate if urease plays a role in facilitating the survival of *C. rodentium* in mice, we compared the ability of the parent strain and its isogenic urease-negative mutant, ICA32, to survive passage through the stomach and colon of mice. To determine the short term effects of gastric acid on bacterial viability, mice were gavaged with the test strains and killed 30 min later, when the bacteria recovered from the stomach and small intestine were enumerated. The results showed that the number of *ureB_CR_* mutant cells (1.6×10^8^ CFU/organ) recovered from these mice 30 min after oral gavage were similar to those recovered from the wild-type strain (1.9×10^8^ CFU per organ; *P*>0.2, Student’s t test), indicating that urease does not enhance the survival of *C. rodentium* during its passage through the mouse stomach.

Three days after inoculation, the numbers of the two strains recovered in the cecum (ICC169, 1.8×10^7^ CFU/organ; ICA32, 1.3×10^6^ CFU/organ), and colon (ICC169, 2.9×10^7^ CFU/organ; ICA32, 1.4×10^7^ CFU/organ) of mice were not significantly different, indicating that the urease-negative mutant was not defective in terms of its ability to survive and replicate *in vivo*.

**Figure 4:**
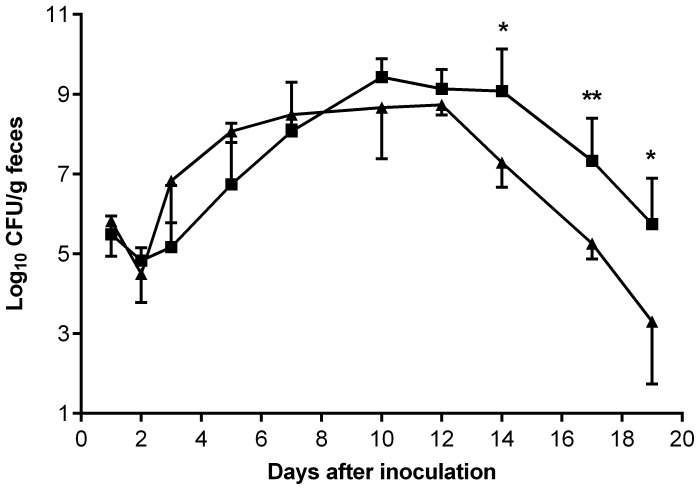
Effect of *ureB* on the ability of *C. rodentium* ICC169 to colonize mice. C57/BL6 mice were inoculated by oral gavage with approximately 10^10^ CFU of wild-type *C. rodentium* (▪) or a *ureB* mutant, ICA32 (▴). Results are the mean ± standard deviation log_10_ CFU/g feces from at least four mice. The limit of detection was 100 CFU/g feces. *, *P*<0.05; **, *P*<0.005, Student’s t test.

To determine if urease contributes to the virulence of *C. rodentium* mice received approximately 1×10^10^ CFU wild-type strain, ICC169, or the urease mutant, ICA32, via oral gavage. Colonization was monitored daily by enumerating bacteria recovered from the feces. When bacterial numbers peaked after inoculation, the mean CFU/g feces of the urease mutant was 3.2×10^9^, which was essentially the same as the maximum mean CFU/g feces of the wild-type strain (4.2×10^9^) (Fig. 4), suggesting that urease does not provide any advantage to medium-term colonization or growth *in vivo*. This study also showed that the numbers of the wild-type and urease mutant strains recovered from the cecum and colon were similar. Although the timing and degree of colonization by mutant ICA32 at the peak of infection did not differ significantly from the wild type, ICA32 appeared to be cleared more efficiently, with the number of CFU of ICA32 recovered from the feces significantly lower than the number of wild-type bacteria recovered from day 14 after inoculation onwards (*P*<0.05, Student’s t test) (Fig. 4).

In all, urease did not appear to enhance the survival of *C. rodentium* during its passage through the stomach or to promote its colonization of the large intestine. However, it may contribute to the ability of *C. rodentium* to persist in the colon. The role that the Pho regulon plays in the induction of the urease gene cluster in *C. rodentium* is unknown, but its effect is probably indirect as no putative Pho boxes are evident upstream of the urease gene cluster.

#### Type III secretory activity and bacterial appendages

The type III secretion system (T3SS) is a highly evolved protein delivery system of Gram-negative bacteria, and can be divided into two major categories: flagellar and virulence-associated. The flagellar T3SS is associated with the MS ring of the basal body and is responsible for secreting the extracytoplasmic structural components of the flagellum [79]. The virulence-associated T3SS is associated with the bacterial injectisome, which delivers effector proteins directly into the cytoplasm of eukaryotic host cells and promotes bacterial infection [80]. Some of the genes encoding flagellar proteins and motor components in *C. rodentium* were down-regulated (*flgBCKL* genes, −2.6- to −1.6-fold) in the *pstCA* mutant when compared to the wild type. FlgB and FlgC are proteins that comprise the rod section of the basal-body assembly of the flagellar motor and are responsible for bacterial locomotion [81], while FlgK and FlgL are hook-filament junction proteins [79]. The *yggR* gene which encodes a protein involved in twitching motility in *Salmonella* Typhimurium was also down regulated (−1.7-fold). However, *C. rodentium* is non-motile and most of the flagellar biosynthesis genes are pseudogenes (e.g., *fliC*, *flgN*, *lfiG*, *lfiI* and *lfgF*) due to insertional inactivation by prophages or insertion elements [8]. It is not clear, therefore, why the *pst* mutation suppresses expression of these genes, but the findings suggest that these genes and pseudogenes may have other functions, e.g., as regulatory elements of *C. rodentium*. It is also possible that PhoB had a regulatory role in the transcription of flagellar genes before these genes became redundant.

Expression of some T3SS genes was also reduced in the *pstCA* mutant by about −1.8-fold. These genes include coding sequences for ORFs ROD29711 and ROD29911, which encode components of the T3SS, and genes for type III-secreted proteins and chaperones such as *nleA*, *nleF*, *nleG8*, *espD*, *espZ* and *cesD2*. Mutations in these genes are only moderately attenuating, in that the mutants retain the ability to colonize mice but induce milder disease than the wild type [82], [83]. NleA, however, does play an important role in the virulence of *C. rodentium* for C3H/HeJ mice, even though it is not required for the formation of A/E lesions and its exact role in infection is unknown [84], [85]. The role of NleG8 *in vivo* has not been studied, although, an NleG homologue of EPEC, named NleI, is translocated into epithelial via the T3SS [86].

**Figure 5:**
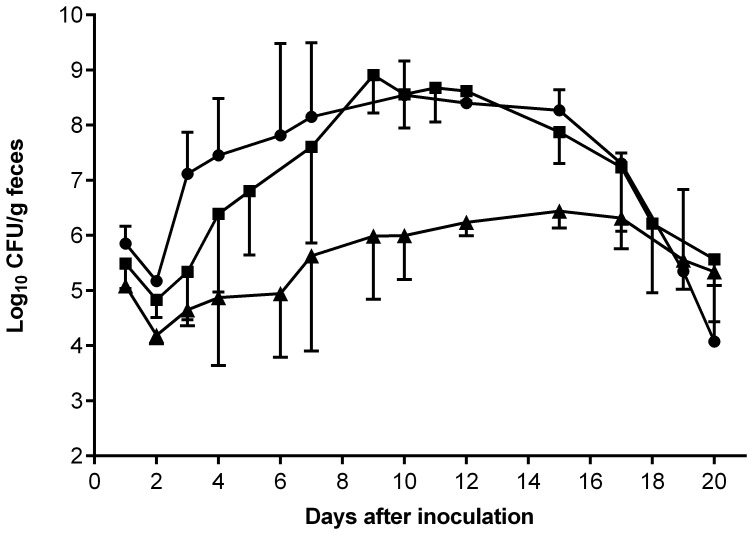
Effect of *nleG8* on the ability of *C. rodentium* to colonize mice. Mice were inoculated by oral gavage with approximately 10^10^ CFU of wild-type *C. rodentium* (▪), a *nleG8* mutant, ICA34 (▴) or a *trans*-complemented mutant ICA34(pAC15) (•). Results are the mean ± standard deviation log_10_ CFU/g feces from at least four mice. The limit of detection was 100 CFU/g feces.

To assess if NleG8 contributes to *C. rodentium* virulence, we constructed a *nleG8* deletion mutant, named ICA34, and a trans-complemented mutant, ICA34(pAC15), and assessed their ability to colonize C57/BL6 mice. Mice inoculated with approximately 1×10^10^ CFU of wild-type ICC169, ICA34 or ICA34(pAC15) via oral gavage showed maximum mean counts of 1.8×10^9^, 5.4×10^6^ and 6.8×10^8^ CFU/g feces, respectively, indicating that *nleG8* contributed to the ability of *C. rodentium* to colonize mice (Fig. 5).

Besides showing reduced T3SS activity, the *pstCA* mutant also exhibited reduced expression (−1.6-fold) of the *tad* locus (for tight, nonspecific adherence). This locus encodes the constituents of a secretion system [87], which is required for the production of long, bundled Flp pili [88], [89]. *tad* is required for the colonization and virulence of *Aggregatibacter actinomycetemcomitans*, *Pasteurella multocida*, *Pseudomonas aeruginosa*, *Yersinia pestis*, *Caulobacter crescentus* and perhaps others [90], [91]. The *tad* locus is arranged as an operon of 14 genes (*flp-1*–*flp-2*–*tadV*– *rcpCAB*–*tadZABCDEFG*), at least twelve of which are required for adherence-related phenotypes, including Flp-pilus production, rough-colony morphology, autoaggregation and biofilm formation [89], [92].

The *flp-1* gene encodes the major component of Flp pili, that belong to the type IVb prepillin family [93], whereas the *tadA* gene product has ATPase activity [94], [95]. RcpA and RcpB are two outer membrane proteins encoded by *rcpA* and *rcpB*, respectively. RcpA is a member of the GspD/PilQ secretin superfamily of proteins found in type II secretion and type IV pilus systems [87], and is predicted to form the outer membrane channel for the secretion of pili [89]. Some of the other Tad components show homology to known bacterial secretion systems, while others, such as RcpB, RcpC, TadZ, TadD and TadG, have no such homology [90]. The chaperone-usher γ4 fimbriae encoded by the ORFs, ROD03641 to ROD03671, were also down-regulated (−2.7-fold). *C. rodentium* carries 19 fimbrial biosynthesis operons. Of these, only colonization factor *Citrobacter* (CFC), a type IV fimbria, and Kfc, a K99-like fimbria [96], are known to play a role in *C. rodentium* virulence [97].

**Figure 6:**
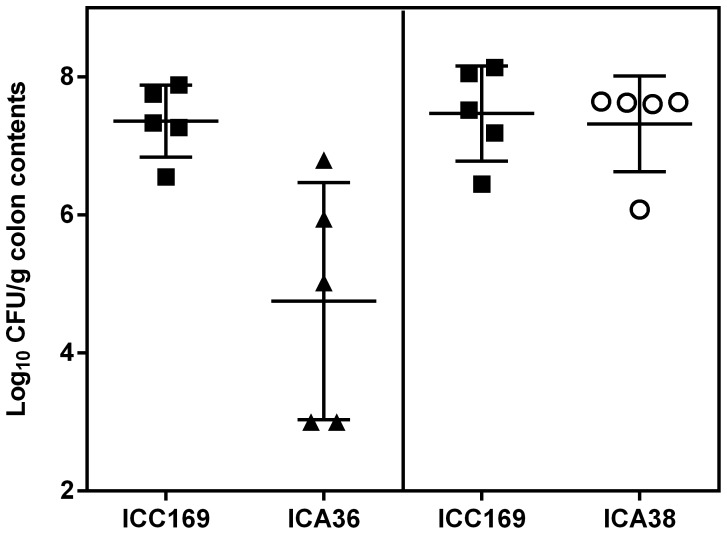
Effect of ROD03671 **and** ***tad*** **on the ability of** ***C. rodentium*** **to colonize mice.** In a competitive infection model, C57/BL6 mice were inoculated by oral gavage with approximately 10^9^ CFU wild-type *C. rodentium* (▪) and either the ROD03671 mutant, ICA36 (▴) or the *tad* mutant, ICA38 (○), in a 1∶1 ratio. Results are the mean ± SD of the numbers of the two strains in the colon contents 7 days after inoculation.

To determine if *C. rodentium* requires the *tad* locus and the chaperone-usher γ4 fimbriae for virulence, site-directed ROD03671 (strain ICA36) and *tad* deletion (ICA38) mutants were constructed, and their virulence was assessed in mice. Experiments in which mice were infected separately with wild type, ROD3671 or *tad* mutants did not show a significant role for either of these loci in mouse colonization (ICC169, maximum mean counts of 1.8×10^9^ CFU/g feces; ROD3671 mutant, 5.4×10^8^ CFU/g feces, and *tad* mutant, 4.8×10^8^ CFU/g feces; *P*>0.5; Student’s t test, 2-tailed). However, mixed-strain infection experiments identified a role for ROD03671 (CI = 0.03), but no role for *tad* (CI = 0.8) in mouse colonization (fig. 6). Taken together, the attenuated virulence of the *pstCA* mutant can be attributed, at least in part, to an overall decrease in the expression of T3SS proteins and adhesins.

### Conclusion

In summary, we compared the gene expression profiles of *C. rodentium* strain ICC169 and its isogenic *pstCA* mutant grown in HPM. In addition to influencing the expression of known Pho regulon genes that play a role in phosphate homeostasis, deletion of *pstCA* resulted in widespread effects, including the induction of a general stress response. We also showed that two of the differentially expressed genes, *degP* and *nleG8*, contribute to the virulence of *C. rodentium*.

Overexpression and mutagenesis studies of *degP* confirmed that this gene is essential for the full virulence of *C. rodentium*. We were, however, unable to complement the phenotype of the *degP* mutant with the wild-type gene. Similar findings have been observed with EPEC strain, E2348/69, where a *degP* mutant was unable to produce bundlin (the structural subunit of BFP), but reintroduction of *degP* on a multicopy complementing plasmid did not restore bundlin synthesis [53], [54]. This could be due to the fact that overproduction of DegP leads to the degradation of protein subunits that are required for the assembly of adhesins, given that the targets of DegP proteolysis in *C. rodentium* are not known. Chaperones and proteases can sequester and eliminate misfolded or damaged proteins, but they may also play a role in salvaging nutrients such as P_i_ from the environment. For example, Vpr, an extracellular serine protease and a putative member of the Pho regulon of *Bacillus subtilis*, is predicted to recover phosphate from phosphoproteins [98] and also to process the peptide antibiotic (lantibiotic) subtilin that targets other Gram-positive bacteria [99], [100]. Conceivably, DegP may play a similar role in *C. rodentium*.

In this study, the non-LEE encoded effector, NleG8, was also differentially expressed in the *pstCA* mutant of *C. rodentium*. Several type III-secreted effector proteins promote bacterial survival and replication by interfering with host cell signaling pathways [101]. Genes encoding these effectors are located within and outside the LEE pathogenicity island. To date, 39 effector proteins in 20 families have been identified in EHEC strain O157:H7, which also harbors the LEE-encoded T3SS [102]. Like EHEC O157:H7, *C. rodentium* encodes a relatively large family of NleG effectors (14 in the EHEC strain with 5 pseudogenes; 5 in *C. rodentium*, with 2 pseudogenes) [102]. Although we showed that *nleG8* is required for the virulence of *C. rodentium*, the secretion and translocation properties, sub-cellular localization and anti-host properties of NleG8 need to be determined.

NleI, a homologue of NleG in EPEC contains secretion and translocation signals at its N-terminus and is translocated into epithelial cells by CesT, a T3SS chaperone [86]. NleI expression is also regulated by SepD at the transcriptional level. Together with SepL, SepD forms a molecular switch that controls the secretion of T3SS translocator proteins [82], [103]. *C. rodentium nleG8* is encoded within a genomic island flanked by insertion sequence elements [8]. Whether this gene is under the control of SepD, PhoB or other regulatory elements is unknown and requires further study. Differential expression of members of the NleG family has also been reported by Bergholz *et al* [104] in their study of EHEC strain O157:H7 grown in low oxygen conditions in minimal medium. Perhaps members of the NleG8 family are also regulated by environmental cues.

As mentioned earlier, cross-regulation can occur between TCRs. For example PhoB activity can be induced in the absence of PhoR by other histidine kinases, such as CreC of the CreC/CreB TCR which is involved in the regulation of genes for carbon catabolism [105]. Thus, activation of some transcriptional factors can affect the expression of genes in other regulons and allows apparently unrelated regulatory networks to respond to the same stimulus [106].

Since both *degP* and *nleG8* play a role in the colonization of *C. rodentium* in mice, we wanted to confirm the participation of the PhoB transcription factor at the promoters of these two target genes. The sequences upstream of *degP* and *nleG8* were inspected for putative PhoB box motifs, but only *degP* contained the putative consensus Pho box sequence (Fig. S1). Although *degP* has not been reported to be directly involved in phosphate metabolism previously [2], preliminary studies have indicated that *phoB* is able to stimulate the transcription of *degP*, albeit at low levels (Table S5), suggesting that the expression of *degP* may be directly affected by PhoB. However, one of two putative Pho boxes located upstream of the *degP* translational start site overlaps with the putative *degP* σ^E^-activatable promoter and is unlikely to be transcriptionally activated by PhoB. Another Pho box which is situated further upstream of the σ^E^ promoter is a more likely candidate. More detailed characterization using Northern blot analysis and gel shift assays to map the transcriptional initiating site of *degP* are needed to confirm direct PhoB-dependent regulation and the exact position of the Pho box. Two other regulons, the Cpx and RpoE (σ^E^) pathways, can also modulate DegP-mediated transcriptional activation [107]. This could in part explain why the increase in the activity of the *degP*-*lacZ* transcriptional fusions mediated by PhoB was lower than expected.

Besides *degP* and *nleG8*, genes encoding several other putative virulence factors were found to be differentially expressed in the *pstCA* mutant. However, two of these putative factors did not contribute to the initial colonization or in vivo growth of *C. rodentium* in C57BL/6 mice. The factors we examined in these animals were a chaperone-usher of γ4 fimbriae and a tight adherence locus. Although the tight adherence locus did not make an obvious contribution to the colonizing ability of *C. rodentium*, it may play a role in autoaggregation and biofilm formation, as in the case of *Actinobacillus*. Also, reduction in the expression of only one these factors may not have a significant impact on the colonizing ability of *C. rodentium*, but they may act in concert so that when expression of more than one is reduced concurrently, such as in a *pstCA* mutant, virulence is diminished. Taken altogether, our results demonstrate the far-reaching and complex regulation by the Pho regulon in *C. rodentium* and provide further evidence that the Pho regulon overlaps with other regulatory pathways, such as RpoS and Cpx, and contributes to bacterial virulence.

## Supporting Information

Figure S1 **Putative Pho box sequence upstream of the** ***degP*** **gene.**
(DOCX)

Table S1 **Oligonucleotide primers used in this study.**
(DOCX)

Table S2 **Genes significantly up-regulated in the** ***pstCA*** **mutant strain compared to wild-type** ***C. rodentium*** **.**
(DOCX)

Table S3 **Genes significantly down-regulated in the** ***pstCA*** **mutant strain compared to wild-type** ***C. rodentium*** **.**
(DOCX)

Table S4 **List of ORFs that were differentially regulated in the** ***pstCA*** **mutant in high phosphate medium.** Only the most significant probe per gene is shown.
(DOCX)

Table S5 **Promoter activity of** ***degP*** **transcriptional fusions in** ***C. rodentium*** **host strains** ***phoB*** **, ICC169 and** ***pstCA*** **cultured in high and low phosphate media, showing the effects of PhoB on levels of expression.**
(DOCX)
