# Supplementary material for: Genome-Wide Analysis of the Pho Regulon in a pstCA Mutant of Citrobacter rodentium
Source: PLoS One. 2012 Nov 30;7(11):e50682. doi: 10.1371/journal.pone.0050682 (PMC3511308; doi:10.1371/journal.pone.0050682)
Supplement: Figure S1 — Putative Pho box sequence upstream of the degP gene. (DOCX) [file pone.0050682.s001.docx]

**Figure S1.** Putative Pho box sequence upstream of the *degP* gene

| Gene |  | Putative Pho box sequence^a^ | | |
| --- | --- | --- | --- | --- |
| Consensus |  | CTGTCAT | A(A/T)A(A/T) | CTGT(CA)A(CT) |
| *degP* | (-65)^b^ | GC**GT**T**AT** | **AAAA**TG**AAT** | **CTG**AGG**T** |
|  | (-93)^b^ | **CT**T**T**TCC | **ATAAA** | **CT**T**TC**G**T** |

*^a^* The bold bases indicate bases identical to the Pho box consensus sequence

*^b^* The position is shown relative to the putative translational start site of *degP.*
