# Supplementary material for: Genome-Wide Analysis of the Pho Regulon in a pstCA Mutant of Citrobacter rodentium
Source: PLoS One. 2012 Nov 30;7(11):e50682. doi: 10.1371/journal.pone.0050682 (PMC3511308; doi:10.1371/journal.pone.0050682)
Supplement: Table S1 — Oligonucleotide primers used in this study. (DOCX) [file pone.0050682.s002.docx]

**Table S1.** Oligonucleotide primers used in this study

| **Primer** | **Sequences (5' to 3')** |
| --- | --- |
| **For RT-PCR** | |
| ICCrrsB-FRT | CAGAGATGCGGTTGTGCCTTCGG |
| ICCrrsB-RRT | CCGCTGGCAACAAAGGATAAGG |
| ICCphoE-FRT | TGGTCGTTAATCTGCGTTTC |
| ICCphoE-RRT | CGATGCACTATTTCAGCGAT |
| ICCytfK-FRT | AACGGTACAACCCACTTCAGGTGG |
| ICCytfK-RRT | ACTTCGGACATCACAGACAAGTGC |
| phoA-FRT2 | CGTCTATCGATAAGCAGGAT |
| phoA-RRT2 | GTAACGATCACCAGCGTATT |
| ICCkatE-FRT | AGCTATCGCACGATGGAAGGATTCG |
| ICCkatE-RRT | TCGTCCCACACCAGCGACGCTTTG |
| ICCmglC-FRT | AATGGCATCCAGCTCATACA |
| ICCmglC-RRT | TGATGATCTATGCGCTCTCC |
| ICClolA-FRT | CAGCTTCACCCAGAAAGTCA |
| ICClolA-RRT | ATGCCAGTTGAACAGATTCG |
| ICCaidB-FRT | CAGCTGTGAGGTGGAGTTTG |
| ICCaidB-RRT | ACAGTCAAAGCGCGTCATAC |
| ICCybdJ-FRT | CATATTCAATCGCGCCAA |
| ICCybdJ-RRT | TGATGGATCTCAACCAGCTT |
| ICCybgA-FRT | ATTCGTGTAATGAGGCGACA |
| ICCybgA-RRT | TCCACAGCCGCTATAAACTG |
| ICCrplV-FRT | CTCAGAAGGTTCGCCTTGTT |
| ICCrplV-RRT | ACCAGTACAGCCGCTTTCTT |
| ICCsucB-FRT | CAACATGAAGCCGATTATGG |
| ICCsucB-RRT | GACATAAAGCCCAGACGGAT |
| ROD03671-FRT | ACCATCAACGGCAAGCTGACGAATG |
| ROD03671-FRT | AGTTGGTTGCGCCCGCGGTGGC |
|  | |
| **For construction of mutants** | |
| pKD4F | TGTGTAGGCTGGAGCTGCTTC |
| pKD4R | CATATGAATATCCTCCTTAG |
| ureBF | AAGACAAGCTGTTGCTCTTTACCG |
| ureBR | ATCGGGATCGTTACGTCAGGCTG |
| ureBkanF | CTAAGGAGGATATTCATATGTCGAGCTGGTGCG |
| ureBkanR | GAAGCAGCTCCAGCCTACACATCGCTGTTGATCG |
| htrAF | TCATCGGCTGGCCTACATTGAGGC |
| htrAR | AGTTCGCCGTATTTGCGCAGATGC |
| htrAkanF | CTAAGGAGGATATTCATATGAGCGTGGCGACAG |
| htrAkanR | GAAGCAGCTCCAGCCTACACAGTTACGCGGCATC |
| ROD03671F | ACTATTTTCTGTCAGGGTCGTTGGC |
| ROD03671R | TCGAAGCGTAATCTCCTTCTCATC |
| ROD03671kanF | CTAAGGAGGATATTCATATGTCCAGATCCAGC |
| ROD03671kanR | GAAGCAGCTCCAGCCTACACAAGCCGGTCAGC |
| nleG8F | ATGATCAGTAGAGAAACTGACAC |
| nleG8R | AGCGCCATCTGTAGCGCATCGCAG |
| nleG8kanF | CTAAGGAGGATATTCATATGATGCACGGTGC |
| nleG8kanR | GAAGCAGCTCCAGCCTACACAACTAAATCCGTC |
| rcpAFF | AGGTCATGTGTTGTATACCTTCCG |
| rcpARR | TGGTAATTCTGCTAACTGACTCGC |
| rcpAkanF | CTAAGGAGGATATTCATATGCTCAAACAGAG |
| rcpAkanR2 | GAAGCAGCTCCAGCCTACACATCGTGACTGACCATG |
|  | |
| **For trans-complementing plasmids** | |
| htrAcF | AGGTCGAGCAGCTTGAGCTACAGG |
| htrAcR | TGGGCCAGCAGGTGCATCAGACGC |
| nleG8cF | TCCATGCCCTCACAAAGAGAGGCTG |
| nleG8cR | TGAGTGGAAGAAGCTTTCCGCACAG |
|  | |
| **For *lacZ* fusion** | |
| htrAforw | AGGATCCGCCTACATTGAGGCGGTAAG |
| htrArev | GAAGCTTAGAACTGCTGGAAGTTACGC |
