# Supplementary material for: Genome-Wide Analysis of the Pho Regulon in a pstCA Mutant of Citrobacter rodentium
Source: PLoS One. 2012 Nov 30;7(11):e50682. doi: 10.1371/journal.pone.0050682 (PMC3511308; doi:10.1371/journal.pone.0050682)
Supplement: Table S2 — Genes significantly up-regulated in the pstCA mutant strain compared to wild-type C. rodentium . (DOCX) [file pone.0050682.s003.docx]

**Table S2.** Genes significantly up-regulated in the *pstCA* mutant strain compared to wild-type *C. rodentium*

| **Functional class and gene name/ORF^a,b^** | **Function** | **Average fold change** |
| --- | --- | --- |
| **Cell envelope** |  |  |
| *yibD*^b^ | Putative glycosyl transferase | 2.18 |
| *ybhT* | Small membrane protein involved in the cell envelope stress response | 1.91 |
| *bcsC* | Cellulose synthase operon protein C | 1.87 |
| *slyB* | Outer membrane lipoprotein | 1.78 |
| *ygaM* | Predicted protein | 1.77 |
| *pagP* | Palmitoyl transferase for lipid A, putative antimicrobial peptide resistance and lipid A acylation | 1.75 |
| *lppA* | Murein lipoprotein | 1.73 |
| *yecN* | Putative inner membrane protein | 1.69 |
| *mipA* | Scaffolding protein for murein synthesizing machinery | 1.59 |
| **Cellular processes** |  |  |
| *katE*^a^ | Catalase HPII | 2.79 |
| *mgrB* | Small protein involved in the acid stress response | 2.39 |
| *bfr*^a^ | Bacterioferritin | 2.06 |
| *cspD*^c^ | Cold shock-like protein | 1.77 |
| *ydhJ*^c^ | Undecaprenyl pyrophosphate phosphatase | 1.72 |
| *maf* | Septum formation protein | 1.54 |
| **Central intermediary metabolism** | | |
| *ppa*^c^ | Inorganic pyrophosphatase | 11.53 |
| *phoA-psiF*^a^ | Alkaline phosphatase | 3.42 |
| *puuD*^c^ | γ-glutamyl-γ-aminobutyrate hydrolase | 1.58 |
| **Energy metabolism** | | |
| *treY* | Malto-oligosyltrehalose synthase | 1.88 |
| *puuB* | γ-glutamylputrescine oxidase | 1.68 |
| *nrfABCDEFG*^e^ | Formate-dependent cytochrome c552/ nitrite reductase | 1.54 |
| **Fatty acid and phospholipid metabolism** | | |
| *cdh* | CDP-diacylglycerol pyrophosphatase | 2.04 |
| **Protein fate** |  |  |
| *tisB* | LexA-regulated toxic peptide | 2.68 |
| *yfiD* | Autonomous glycyl radical cofactor | 2.52 |
| *degP*^d^ | Periplasmic serine protease | 2.46 |
| ROD29711 | T3SS component | 2.21 |
| *clpS*^c^ | ATP-dependent Clp protease adaptor protein | 1.83 |
| *tatE* | Twin arginine translocase protein E | 1.68 |
| *iraP* | Anti-adaptor protein for σ^s^ stabilisation | 1.53 |
| **Transcription** |  |  |
| *phoBR*^b,c^ | DNA-binding response regulator in two-component regulatory system with PhoR (or CreC) | 4.32 |
| *rraA* | Regulator of ribonuclease activity A | 2.38 |
| *yfeD* | Predicted DNA-binding transcriptional regulator | 1.84 |
| *bssR*^c^ | Biofilm regulator | 1.83 |
| *rpoS* | RNA polymerase sigma factor | 1.77 |
| *caiF* | DNA-binding transcriptional activator of carnitine metabolism | 1.72 |
| *dksA* | RNA polymerase-binding transcription factor | 1.65 |
| *mlrA* | DNA-binding transcriptional regulator of curli and extracellular matrix synthesis | 1.63 |
| *rcsB* | DNA-binding transcriptional regulator for colanic capsule biosynthesis | 1.57 |
| **Transport and binding proteins** | | |
| *phoE*^b,c^ | Outer membrane phosphoporin protein E | 7.75 |
| *ugpBAECQ*^a,b,c^ | Glycerol-3-phosphate transport system | 4.81 |
| *pstSCAB-phoU*^b,c^ | High-affinity phosphate ABC transport system | 3.62 |
| *gntX* | Gluconate periplasmic binding protein | 3.33 |
| *rcnA* | Nickel/cobalt efflux system | 2.30 |
| *yjeP*^d^ | Predicted mechanosensitive channel | 1.77 |
| *artJ*^a,c^ | Arginine-binding periplasmic protein 2 | 1.74 |
| *ppx*^c^ | Exopolyphosphatase | 1.65 |
| *ftnA* | Ferritin iron storage protein | 1.63 |
| *cutC* | Copper homeostasis protein | 1.55 |
| *sapF* | Predicted antimicrobial peptide transporter subunit | 1.51 |
| **Unknown function** |  |  |
| *ygiW*^a^ | Conserved outer membrane protein | 5.70 |
| *ytfK*^a,b^ | Conserved protein | 4.91 |
| ROD23211 | Small hypothetical protein | 3.89 |
| ROD05111 | Hypothetical protein | 3.29 |
| ROD19811 | Hypothetical protein | 2.53 |
| ROD05801 | Putative small toxic membrane polypeptide | 2.49 |
| *ycfP* | Conserved protein | 2.46 |
| ROD09131 | Hypothetical protein | 2.46 |
| *yjdJ*^a^ | Predicted acyltransferase with acyl-CoA N-acyltransferase domain | 2.44 |
| ROD14971 | Small hypothetical protein | 2.38 |
| ROD36211 | Hypothetical protein | 2.35 |
| *yodC*^a^ | Hypothetical protein | 2.27 |
| *ycfP* | Conserved protein | 2.27 |
| *phoH*^b,c^ | Putative phosphate starvation-inducible protein | 2.23 |
| ROD36481 | Hypothetical protein | 2.22 |
| ROD23581 | Hypothetical protein | 2.11 |
| ROD17581 | Hypothetical protein | 2.09 |
| ROD26221 | Hypothetical protein | 2.08 |
| *yeaQ* | Conserved inner membrane protein | 2.06 |
| *chbG* | Conserved protein | 2.05 |
| ROD20361 | Hypothetical protein | 1.91 |
| *ybeD* | Conserved protein required for swarming phenotype | 1.90 |
| *yeeD* | Conserved protein | 1.56 |
| *mraZ* | Conserved protein | 1.56 |
| *yeaL*^c^ | Conserved inner membrane protein | 1.56 |
| *yodC*^a^ | Hypothetical protein | 1.56 |
| *yggE*^a^ | Putative immunogenic protein | 1.53 |
| **Biosynthesis of cofactors, prosthetic groups, and carriers** | | |
| *cybB* | Cytochrome b561 | 2.55 |
| *ynfK* | Putative dethiobiotin synthetase | 1.66 |
| *nudB* | Dihydroneopterin triphosphate pyrophosphohydrolase | 1.56 |
| **Mobile and extrachromosomal element functions** | | |
| ROD02521 | Predicted tail sheath protein | 2.97 |
| ROD25761 | Hypothetical prophage protein | 2.70 |
| *ymfH* | e14 prophage | 2.30 |
| ROD25891 | Bacteriophage tail tube protein | 2.23 |
| ROD25951 | Phage major capsid protein E | 2.12 |

^a^ Genes belonging to the RpoS regulon

^b^ Genes belonging to the Pho regulon

^c^ Indicates the presence of a putative Pho box in the gene promoter

^d^ Genes belonging to the Cpx regulon

^e^ Genes belonging to the c-AMP receptor protein (CRP) regulon

^f^ Genes shown in boldface are part of an operon in which two or more genes are up-regulated in the *pstCA* mutant.
