# Supplementary material for: Genome-Wide Analysis of the Pho Regulon in a pstCA Mutant of Citrobacter rodentium
Source: PLoS One. 2012 Nov 30;7(11):e50682. doi: 10.1371/journal.pone.0050682 (PMC3511308; doi:10.1371/journal.pone.0050682)
Supplement: Table S3 — Genes significantly down-regulated in the pstCA mutant strain compared to wild-type C. rodentium . (DOCX) [file pone.0050682.s004.docx]

**Table S3.** Genes significantly down-regulated in the *pstCA* mutant strain compared to wild-type *C. rodentium*

| **Functional class and gene name/ORF^a,b^** | **Function** | **Average fold change** |
| --- | --- | --- |
| **Cell envelope** |  |  |
| ROD03671 | Putative F17-like fimbrial subunit, chaperone-usher γ4 fimbriae | -2.68 |
| *ompA*^c,e^ | Outer membrane protein A | -1.88 |
| *srfA* | T3SS translocator protein | -1.76 |
| ROD15641 | Outer membrane porin protein | -1.70 |
| *ompF*^c,d,e^ | Outer membrane protein F | -1.69 |
| *rcpA* | Putative virulence effector protein, *ssrAB* activated gene | -1.58 |
| *espD* | T3SS translocator protein | -1.54 |
| **Cellular processes** | | |
| *flgBCKL* | Flagellar basal body rod and hook associated protein | -2.57 |
| *sodA* | Manganese superoxide dismutase | -1.66 |
| *ftsY* | Cell division membrane protein | -1.62 |
| *yggR* | Predicted transporter | -1.59 |
| *yjiO* | Multidrug efflux system protein | -1.57 |
| *lafD* | Lateral flagellar chaperone protein | -1.54 |
| **Amino acid biosynthesis** | | |
| *serA* | D-3-phosphoglycerate dehydrogenase | -2.69 |
| *gltB* | Glutamate synthase | -1.92 |
| *metE* | Cobalamin-independent homocysteine transmethylase | -1.85 |
| *glnA*^c^ | Glutamine synthetase | -1.85 |
| *argE*^c^ | Acetylornithine deacetylase | -1.68 |
| *mlr* | Putative selenocysteine synthase | -1.63 |
| *aroC* | Chorismate synthase | -1.54 |
| *hisB* | Histidinol-phosphatase | -1.52 |
| **Central intermediary metabolism** | | |
| *ureDABEFG* | Urease accessory protein, urease gamma and beta subunit | -3.44 |
| *hycABCDEF*^a^*GHI* | Formate hydrogenlyase | -1.83 |
| *gldA* | Glycerol dehydrogenase | -1.63 |
| *ampG*^c^ | Muropeptide transporter, permease involved in peptidoglycan recycling | -1.55 |
| **Energy metabolism** | | |
| *glpE* | Thiosulfate sulphur-transferase | -2.55 |
| *eutC* | Ethanolamine ammonia-lyase | -2.22 |
| *sucAB*^c^*C*^c^*D* | Dihydrolipoamide succinyltransferase | -2.03 |
| *tktA^c^* | Transketolase | -1.90 |
| *xylB* | Xylulokinase | -1.83 |
| *dsdA* | D-serine deaminase | -1.79 |
| *acnB*^a,e^ | Aconitate hydratase 2 CRP | -1.78 |
| *cyoA*^c^*^,^*^e^*BCDE* | Cytochrome o ubiquinol oxidase C-reactive protein | -1.76 |
| *gltA*^c^ | Type II citrate synthase | -1.75 |
| *sdhC*^c^*DA*^c^*B* | Succinate dehydrogenase flavoprotein | -1.71 |
| *dmsABC* | Dimethyl sulfoxide reductase | -1.69 |
| *dgoRKADT* | 2-dehydro-3-deoxy-6-phosphogalactonate aldolase | -1.68 |
| *aceF* | Pyruvate dehydrogenase | -1.65 |
| *atpBEFHAGDC* | ATP synthase F1 | -1.64 |
| *lldD* | L-lactate dehydrogenase | -1.59 |
| *tdcD* | Propionate kinase | -1.59 |
| *tdh* | L-threonine 3-dehydrogenase | -1.58 |
| *argI^c^* | Ornithine carbamoyltransferase 1 | -1.56 |
| *gcvT* | Aminomethyltransferase T | -1.56 |
| *garK* | Glycerate kinase I | -1.54 |
| *gatD*^e^ | Galactitol-1-phosphate dehydrogenase | -1.53 |
| *pckA* | Phosphoenolpyruvate carboxykinase | -1.52 |
| *glpK* | Glycerol kinase | -1.51 |
| **Fatty acid and phospholipid metabolism** | |  |
| *fabI* | Enoyl-(acyl carrier protein)-reductase | -1.57 |
| *accB*^c^ | Biotin carboxyl carrier protein of acetyl-coA carboxylase | -1.56 |
| **Protein fate** |  |  |
| *espZ/sepZ* | T3SS secreted effector protein | -2.61 |
| *nleF* | T3SS secreted effector protein | -1.85 |
| ROD29711 | T3SS component | -1.82 |
| *cesD2* | T3SS chaperone | -1.68 |
| *dsbC* | Protein disulfide isomerase II | -1.65 |
| *nleA* | T3SS secreted effector protein | -1.65 |
| ROD29691 | LEE-associated conserved hypothetical protein | -1.62 |
| ROD17861 | Putative invasin/intimin-like protein | -1.59 |
| *nleG8* | T3SS secreted effector protein | -1.59 |
| ROD29911 | T3SS component | -1.54 |
| *yegQ* | Putative protease | -1.51 |
|  |  |  |
| **Protein synthesis** |  |  |
| *rpsJ-rplC*^c^*DWB-rpsS-rplV-rpsC-rplP-rpmC-rpsQ* | 50S ribosomal protein L23 | -1.85 |
| *rplNXE-rpsN*^c^*H-rplFR-rpsE-rpmD-rplO-secY-rpmJ* | 50S ribosomal protein L18 | -1.81 |
| *infA*^a^ | Translation initiation factor IF-1 | -1.65 |
| *infB* | Translation initiation factor IF-2 | -1.58 |
| *lexA* | Regulator for SOS regulon | -1.56 |
| *rbn* | tRNA-processing ribonuclease | -1.54 |
| *metG*^c^ | Methionyl-tRNA synthetase | -1.53 |
| *rpsMKD*^c^*-rpoA*^c^*-rplQ* | 30S ribosomal protein S11 | -1.53 |
| **Purines, pyrimidines, nucleosides, and nucleotides** | |  |
| *carB* | Carbamoyl-phosphate synthase | -1.58 |
| **Transcription** |  |  |
| *rhlB*^e^ | ATP-dependent RNA helicase | -1.68 |
| ROD41191 | Putative LacI-family transcriptional regulator | -1.56 |
| ROD43621 | Gntr-family transcriptional regulator | -1.50 |
| **Regulatory function** | | |
| *cdaR* | Carbohydrate diacid regulator | -1.70 |
| *cspA*^c^ | Major cold shock protein | -1.54 |
| **Transport and binding proteins** | | |
| *yhjX* | Putative transporter | -1.84 |
| *zraP*^e^ | Zinc homeostasis protein | -1.83 |
| *proW*^c^ | Glycine betaine transporter, permease protein | -1.77 |
| *gltK* | Glutamate/aspartate transport protein | -1.74 |
| *manY* | Mannose-specific phosphotransferase enzyme II | -1.72 |
| *ompN* | Outer membrane pore protein N | -1.66 |
| *emrD*^c^ | Multidrug resistance protein D | -1.65 |
| *malF* | Maltose transport protein | -1.61 |
| ROD12431 | D-galactonate transporter | -1.61 |
| *yhjE* | Putative metabolite transport protein | -1.61 |
| *dppABCDF* | Dipeptide transport protein | -1.58 |
| *Nepl* | Purine ribonucleoside transporter | -1.56 |
| *gntU* | Low-affinity gluconate transporter | -1.55 |
| *livKHMGF* | Leucine-specific binding protein | -1.52 |
| *gntT* | High-affinity gluconate transporter | -1.51 |
| *pcaK* | Hydroxybenzoate transporter | -1.51 |

| **Unknown function** |  |  |
| --- | --- | --- |
| ROD36321 | Putative lipoprotein | -1.87 |
| ROD33021 | Putative inner membrane protein | -1.75 |
| ROD03691 | Colicin V precursor | -1.75 |
| ROD32991 | Putative cytoplasmic protein | -1.64 |
| *yhbVW*^c^ | Putative monooxygenase | -1.57 |
| *ybgA*^a^ | Putative pathogenicity island protein | -1.57 |
| ROD14011 | Glyoxalase/bleomycin resistance protein/dioxygenase | -1.54 |
| ROD40931 | Hypothetical protein | -1.53 |
| *yceG* | Predicted aminodeoxychorismate lyase | -1.51 |
| **Biosynthesis of cofactors, prosthetic groups, and carriers** | |  |
| *thiG* | Thiamine biosynthesis protein | -1.70 |
| **Mobile and extrachromosomal element function** | |  |
| ROD36231-51 | Iscro1 transposase ABC | -1.87 |
| ROD36691 | Putative prophage lipoprotein | -1.67 |
| ROD38021 | Transposase, IS110 family | -1.64 |
| ROD01351 | Transposase, IS110 family | -1.63 |
| ROD05781 | Putative transposase | -1.61 |
| ROD47321 | Putative phage baseplate protein | -1.61 |
| ROD47311 | Phage DNA circularisation protein | -1.56 |

^a^ Genes belonging to the RpoS regulon

^b^ Genes belonging to the Pho regulon

^c^ Indicates the presence of a putative Pho box in the gene promoter

^d^ Genes belonging to the Cpx regulon

^e^ Genes belonging to the c-AMP receptor protein (CRP) regulon

^f^ Genes shown in boldface are part of an operon in which two or more genes are down-regulated in the *pstCA* mutant
