# Supplementary material for: Genome-Wide Analysis of the Pho Regulon in a pstCA Mutant of Citrobacter rodentium
Source: PLoS One. 2012 Nov 30;7(11):e50682. doi: 10.1371/journal.pone.0050682 (PMC3511308; doi:10.1371/journal.pone.0050682)
Supplement: Table S4 — List of ORFs that were differentially regulated in the pstCA mutant in high phosphate medium. Only the most significant probe per gene is shown. (DOCX) [file pone.0050682.s005.docx]

**Table S4.** List of ORFs that were differentially regulated in the *pstCA* mutant in high phosphate medium. Only the most significant probe per gene is shown.

| **Up-regulated** | | |
| --- | --- | --- |
| **ORF** | **Fold change** | **Adjusted P value** |
| ROD00371 | 1.72 | 0.000419 |
| ROD00661 | 1.62 | 0.00185 |
| ROD00871 | 1.64 | 0.00188 |
| ROD01491 | 1.65 | 0.000308 |
| ROD01671 | 2.20 | 0.00121 |
| ROD02391 | 2.90 | 2.70x10^-05^ |
| ROD02501 | 2.34 | 0.00109 |
| ROD02521 | 2.97 | 0.000317 |
| ROD02531 | 2.13 | 0.00109 |
| ROD02551 | 2.39 | 0.00177 |
| ROD02561 | 1.97 | 0.00129 |
| ROD02571 | 2.53 | 0.00144 |
| ROD02631 | 1.52 | 0.00628 |
| ROD02641 | 2.43 | 0.00182 |
| ROD02671 | 2.85 | 0.00139 |
| ROD02681 | 2.62 | 0.000803 |
| ROD03361 | 7.75 | 6.53x10^-12^ |
| ROD04271 | 3.42 | 1.79x10^-06^ |
| ROD04281 | 3.46 | 4.12x10^-06^ |
| ROD04361 | 1.63 | 0.00268 |
| ROD04431 | 4.32 | 4.12x10^-06^ |
| ROD04441 | 3.74 | 1.17 x10^-08^ |
| ROD05111 | 2.88 | 0.000124 |
| ROD05361 | 1.52 | 0.000367 |
| ROD05381 | 1.52 | 0.00151 |
| ROD05801 | 2.49 | 0.000523 |
| ROD06371 | 1.75 | 0.00134 |
| ROD06411 | 1.68 | 0.00181 |
| ROD06491 | 1.90 | 0.000739 |
| ROD07051 | 1.61 | 0.00294 |
| ROD07581 | 1.91 | 0.000662 |
| ROD08391 | 1.83 | 0.00636 |
| ROD08911 | 1.77 | 0.00331 |
| ROD08921 | 1.83 | 0.00274 |
| ROD09012 | 1.52 | 0.00145 |
| ROD09131 | 2.46 | 0.000207 |
| ROD09141 | 2.28 | 0.000349 |
| ROD09151 | 1.55 | 0.00123 |
| ROD09161 | 1.78 | 0.00107 |
| ROD09201 | 1.87 | 0.00115 |
| ROD09211 | 2.19 | 0.000648 |
| ROD09601 | 1.72 | 0.00547 |
| ROD10281 | 1.61 | 0.00188 |
| ROD10741 | 2.85 | 0.000155 |
| ROD10751 | 1.55 | 0.0455 |
| ROD11041 | 1.54 | 0.00252 |
| ROD11491 | 2.46 | 4.12 x10^-06^ |
| ROD11701 | 1.53 | 0.00706 |
| ROD12571 | 2.06 | 0.00174 |
| ROD12651 | 1.59 | 0.00121 |
| ROD12701 | 2.05 | 5.95 x10^-05^ |
| ROD13231 | 2.79 | 2.37 x10^-06^ |
| ROD13611 | 1.58 | 0.000853 |
| ROD13731 | 1.73 | 0.00388 |
| ROD13961 | 1.67 | 0.00110 |
| ROD14161 | 1.55 | 0.00227 |
| ROD14241 | 1.72 | 0.00117 |
| ROD14261 | 1.78 | 7.15 x10^-05^ |
| ROD14561 | 1.54 | 0.00318 |
| ROD14721 | 1.66 | 0.0163 |
| ROD14961 | 1.91 | 0.00145 |
| ROD14971 | 2.38 | 0.000648 |
| ROD15361 | 1.63 | 0.000282 |
| ROD15591 | 11.53 | 1.19 x10^-09^ |
| ROD16211 | 1.55 | 0.00275 |
| ROD16601 | 1.84 | 0.0131 |
| ROD17101 | 1.51 | 0.00135 |
| ROD17261 | 1.58 | 0.00832 |
| ROD17581 | 2.09 | 0.000535 |
| ROD17941 | 1.82 | 0.00949 |
| ROD18081 | 1.89 | 0.00642 |
| ROD18641 | 1.62 | 0.00435 |
| ROD18651 | 2.39 | 0.000246 |
| ROD19061 | 1.56 | 0.00254 |
| ROD19151 | 1.69 | 0.000618 |
| ROD19181 | 1.55 | 0.00233 |
| ROD19461 | 1.63 | 0.00126 |
| ROD19601 | 1.50 | 0.0313 |
| ROD19651 | 1.93 | 5.78 x10^-05^ |
| ROD19801 | 1.91 | 0.00240 |
| ROD19811 | 2.50 | 0.000367 |
| ROD20361 | 1.82 | 0.000532 |
| ROD21511 | 1.56 | 0.00155 |
| ROD22291 | 1.89 | 0.00157 |
| ROD22801 | 1.57 | 0.00182 |
| ROD23211 | 4.11 | 9.47 x10^-05^ |
| ROD23501 | 1.63 | 0.00233 |
| ROD23581 | 1.93 | 1.68 x10^-05^ |
| ROD24411 | 1.52 | 0.00604 |
| ROD24471 | 1.60 | 0.00275 |
| ROD25241 | 2.70 | 0.00502 |
| ROD25752 | 1.72 | 0.00391 |
| ROD25761 | 2.23 | 0.00157 |
| ROD25861 | 1.71 | 0.000200 |
| ROD25891 | 1.56 | 0.000622 |
| ROD25901 | 1.57 | 0.000132 |
| ROD25941 | 2.08 | 0.000157 |
| ROD25951 | 2.13 | 0.000122 |
| ROD26221 | 1.80 | 0.00107 |
| ROD26222 | 3.74 | 9.72 x10^-05^ |
| ROD26223 | 2.30 | 0.000193 |
| ROD28171 | 1.62 | 1.62 x10^-05^ |
| ROD29021 | 2.05 | 0.000303 |
| ROD29031 | 1.64 | 0.00121 |
| ROD29041 | 2.21 | 0.000523 |
| ROD29051 | 1.77 | 0.000432 |
| ROD29531 | 1.77 | 0.00493 |
| ROD29711 | 1.72 | 0.00154 |
| ROD31361 | 1.51 | 0.00624 |
| ROD32211 | 1.71 | 0.00104 |
| ROD35271 | 1.84 | 0.00157 |
| ROD35661 | 1.52 | 0.00446 |
| ROD36211 | 2.39 | 0.000369 |
| ROD36481 | 2.37 | 0.000894 |
| ROD38171 | 1.53 | 0.00233 |
| ROD38681 | 1.51 | 0.00109 |
| ROD40131 | 2.70 | 7.70 x10^-05^ |
| ROD40161 | 2.68 | 5.23 x10^-09^ |
| ROD40171 | 2.88 | 6.91 x10^-08^ |
| ROD40581 | 2.13 | 4.64 x10^-06^ |
| ROD42771 | 1.51 | 0.0197 |
| ROD43071 | 1.81 | 0.00129 |
| ROD43751 | 3.33 | 4.10 x10^-07^ |
| ROD43781 | 2.06 | 4.12 x10^-06^ |
| ROD44101 | 1.63 | 0.00435 |
| ROD44861 | 1.60 | 0.00255 |
| ROD45101 | 1.53 | 0.00554 |
| ROD46211 | 1.62 | 0.000679 |
| ROD50771 | 1.54 | 0.000552 |

| **Down-regulated** | | |
| --- | --- | --- |
| **ORF** | **Fold change** | **Adjusted P value** |
| ROD00281 | -1.53 | 0.00475 |
| ROD01211 | -1.65 | 0.00227 |
| ROD01241 | -1.58 | 0.00348 |
| ROD01251 | -1.78 | 0.00333 |
| ROD01351 | -1.63 | 0.00130 |
| ROD01661 | -1.70 | 0.00156 |
| ROD02021 | -1.66 | 0.00233 |
| ROD02771 | -1.54 | 0.00417 |
| ROD03201 | -1.55 | 0.00365 |
| ROD03241 | -1.51 | 0.00305 |
| ROD03571 | -1.58 | 0.00195 |
| ROD03671 | -2.68 | 0.000157 |
| ROD03681 | -1.99 | 5.17 x10^-06^ |
| ROD03691 | -1.75 | 3.38 x10^-05^ |
| ROD04841 | -1.55 | 0.000877 |
| ROD04871 | -1.66 | 0.00174 |
| ROD04881 | -1.55 | 0.000694 |
| ROD05781 | -1.61 | 0.00123 |
| ROD06001 | -1.76 | 0.0174 |
| ROD07181 | -1.75 | 0.00218 |
| ROD07191 | -1.71 | 0.000991 |
| ROD07211 | -1.60 | 0.00523 |
| ROD07241 | -2.03 | 0.00326 |
| ROD07251 | -1.63 | 0.00910 |
| ROD07691 | -1.84 | 0.0306 |
| ROD07991 | -1.55 | 0.00369 |
| ROD08131 | -1.96 | 0.00397 |
| ROD08991 | -1.74 | 0.00275 |
| ROD09485 | -1.71 | 0.0113 |
| ROD09981 | -1.65 | 0.000253 |
| ROD10021 | -1.69 | 0.00987 |
| ROD10191 | -1.57 | 0.00973 |
| ROD10801 | -1.88 | 0.00449 |
| ROD11401 | -1.76 | 0.0256 |
| ROD11411 | -2.57 | 0.00781 |
| ROD11421 | -2.40 | 0.00678 |
| ROD11431 | -1.66 | 0.00455 |
| ROD11591 | -1.66 | 0.00123 |
| ROD12201 | -1.51 | 0.00234 |
| ROD12431 | -1.61 | 0.00338 |
| ROD13241 | -1.61 | 0.00450 |
| ROD13711 | -1.73 | 0.00517 |
| ROD13741 | -1.51 | 0.0102 |
| ROD14011 | -1.54 | 0.00144 |
| ROD14621 | -1.78 | 0.00547 |
| ROD14631 | -1.59 | 0.00501 |
| ROD15641 | -1.70 | 0.0260 |
| ROD15861 | -1.58 | 0.00335 |
| ROD17861 | -1.59 | 0.0413 |
| ROD18561 | -1.72 | 0.00195 |
| ROD18951 | -1.93 | 0.00389 |
| ROD20161 | -1.82 | 0.00705 |
| ROD20171 | -1.56 | 0.0478 |
| ROD20241 | -1.71 | 0.00426 |
| ROD21311 | -1.65 | 0.0456 |
| ROD21601 | -1.52 | 0.00109 |
| ROD22061 | -1.53 | 0.0157 |
| ROD22131 | -1.51 | 0.00128 |
| ROD23401 | -1.61 | 0.00557 |
| ROD23971 | -2.22 | 0.00270 |
| ROD24911 | -1.61 | 0.0108 |
| ROD25471 | -1.51 | 0.00487 |
| ROD27411 | -1.54 | 0.00215 |
| ROD29422 | -1.68 | 0.00275 |
| ROD29691 | -1.62 | 0.00364 |
| ROD29711 | -1.82 | 0.001539 |
| ROD29731 | -1.68 | 0.00575 |
| ROD29751 | -1.54 | 0.0222 |
| ROD29911 | -1.54 | 0.00416 |
| ROD29961 | -2.61 | 0.000188 |
| ROD30871 | -1.51 | 0.0199 |
| ROD30911 | -1.83 | 0.00317 |
| ROD31051 | -1.52 | 0.0221 |
| ROD31461 | -1.54 | 0.0181 |
| ROD31471 | -1.58 | 0.0395 |
| ROD31501 | -1.50 | 0.00387 |
| ROD31751 | -1.66 | 0.00217 |
| ROD31831 | -1.63 | 0.00664 |
| ROD32571 | -1.52 | 0.0395 |
| ROD32781 | -1.77 | 0.00275 |
| ROD32981 | -1.50 | 0.000467 |
| ROD32991 | -1.64 | 0.000498 |
| ROD33021 | -1.83 | 0.00135 |
| ROD33051 | -1.92 | 0.000181 |
| ROD33201 | -1.56 | 0.000419 |
| ROD33601 | -1.82 | 0.00154 |
| ROD33661 | -1.64 | 0.00355 |
| ROD34421 | -1.55 | 0.00260 |
| ROD34601 | -1.54 | 0.00708 |
| ROD34611 | -1.54 | 0.0210 |
| ROD34631 | -1.51 | 0.00658 |
| ROD34921 | -1.69 | 0.00294 |
| ROD34971 | -1.51 | 0.00519 |
| ROD35121 | -1.56 | 0.00293 |
| ROD36231 | -1.75 | 0.00493 |
| ROD36251 | -1.55 | 0.00403 |
| ROD36271 | -1.55 | 0.00295 |
| ROD36321 | -1.65 | 0.00227 |
| ROD36691 | -1.67 | 0.00258 |
| ROD36831 | -1.58 | 0.00335 |
| ROD37121 | -1.61 | 0.00330 |
| ROD37501 | -1.84 | 0.00157 |
| ROD37621 | -1.70 | 0.00761 |
| ROD37881 | -1.68 | 0.000118 |
| ROD37991 | -1.63 | 0.00633 |
| ROD38021 | -1.64 | 0.00143 |
| ROD38221 | -1.51 | 0.00108 |
| ROD38431 | -1.66 | 0.00395 |
| ROD38741 | -1.54 | 0.00268 |
| ROD38911 | -1.85 | 0.00260 |
| ROD39251 | -1.85 | 0.00395 |
| ROD39671 | -1.68 | 0.00102 |
| ROD40051 | -1.61 | 0.0115 |
| ROD40071 | -1.64 | 0.00552 |
| ROD40121 | -2.00 | 0.00342 |
| ROD40321 | -1.68 | 0.00219 |
| ROD40531 | -1.79 | 0.00145 |
| ROD40561 | -1.65 | 0.00376 |
| ROD40931 | -1.53 | 0.000635 |
| ROD40971 | -1.55 | 0.000498 |
| ROD41131 | -1.56 | 0.00155 |
| ROD41191 | -1.56 | 0.00188 |
| ROD41211 | -1.53 | 0.00326 |
| ROD41401 | -1.60 | 0.00459 |
| ROD41801 | -2.00 | 0.00369 |
| ROD42001 | -1.58 | 0.00267 |
| ROD42151 | -1.59 | 0.00180 |
| ROD42421 | -1.83 | 0.00217 |
| ROD42491 | -1.54 | 0.000182 |
| ROD42511 | -1.51 | 0.00220 |
| ROD42601 | -1.84 | 0.00321 |
| ROD42631 | -1.61 | 0.00411 |
| ROD43571 | -1.62 | 0.00267 |
| ROD43621 | -1.50 | 0.00052 |
| ROD43681 | -1.52 | 0.00130 |
| ROD43901 | -1.55 | 0.00275 |
| ROD44081 | -1.51 | 0.00238 |
| ROD44211 | -1.53 | 0.000164 |
| ROD44601 | -1.69 | 0.0226 |
| ROD44741 | -1.55 | 0.00291 |
| ROD45021 | -1.75 | 0.0116 |
| ROD45031 | -1.81 | 0.0119 |
| ROD45051 | -1.79 | 0.00521 |
| ROD45061 | -1.85 | 0.00801 |
| ROD45071 | -1.74 | 0.00416 |
| ROD45081 | -1.59 | 0.0170 |
| ROD45161 | -1.70 | 0.0147 |
| ROD45181 | -1.60 | 0.032 |
| ROD45201 | -1.52 | 0.0265 |
| ROD45241 | -1.53 | 0.0231 |
| ROD45591 | -1.56 | 0.00366 |
| ROD45871 | -1.50 | 0.0132 |
| ROD46051 | -1.62 | 0.00609 |
| ROD46501 | -1.53 | 0.00374 |
| ROD46601 | -1.63 | 0.00122 |
| ROD46641 | -1.58 | 0.000793 |
| ROD46721 | -1.57 | 0.00188 |
| ROD46931 | -1.60 | 0.00368 |
| ROD47311 | -1.56 | 0.00196 |
| ROD47321 | -1.61 | 0.002595 |
| ROD47462 | -1.53 | 0.00351 |
| ROD47551 | -1.54 | 0.00145 |
| ROD47621 | -1.59 | 0.00241 |
| ROD47661 | -1.54 | 0.00367 |
| ROD47741 | -1.87 | 0.0129 |
| ROD48291 | -1.57 | 0.00179 |
| ROD48871 | -1.55 | 0.00155 |
| ROD49181 | -2.69 | 3.38 x10^-05^ |
| ROD49281 | -1.56 | 0.000432 |
| ROD49401 | -1.65 | 0.00157 |
| ROD49831 | -1.56 | 0.00477 |
| ROD49851 | -1.58 | 0.00322 |
| ROD50041 | -1.71 | 0.00362 |
| ROD50121 | -1.66 | 0.00829 |
| ROD50511 | -1.90 | 0.00233 |
| ROD50641 | -1.57 | 0.0158 |
