# Supplementary material for: Genome-Wide Analysis of the Pho Regulon in a pstCA Mutant of Citrobacter rodentium
Source: PLoS One. 2012 Nov 30;7(11):e50682. doi: 10.1371/journal.pone.0050682 (PMC3511308; doi:10.1371/journal.pone.0050682)
Supplement: Table S5 — Promoter activity of degP transcriptional fusions in C. rodentium host strains phoB , ICC169 and pstCA cultured in high and low phosphate media, showing the effects of PhoB on levels of expression. (DOCX) [file pone.0050682.s006.docx]

**Table S5.** Promoter activity of *degP* transcriptional fusions in *C. rodentium* host strains *phoB*, ICC169 and *pstCA* cultured in high and low phosphate media, showing the effects of PhoB on levels of expression.

| Transcriptional fusion | Culture medium | | | | | |
| --- | --- | --- | --- | --- | --- | --- |
|  | MOPS with high Pi | | | MOPS with low Pi | | |
|  | ∆*phoBkan*^a^ (PhoB^−^) | ICC169 (PhoB^+^) | ∆*pstCA* (PhoB^+^) | ∆*phoBkan* (PhoB^−^) | ICC169 (PhoB^+^) | ∆*pstCA* (PhoB^+^) |
| *degP-lacZ* | 110 ^b^ | 104 (0.95) | 214 (1.95) | 118 | 210 (1.78) | 224 (1.90) |

^a^ Characteristics of test strains, all of which were derived from the *C. rodentium* ICC169 wild type

^b^ Specific activity of β-galactosidase (Miller units). Data are the mean of three independent assays in which samples were tested in duplicate, and the standard deviations were less than 15%. The numbers in parentheses indicate the fold activation, which is the ratio of the specific activity of β-galactosidase of the PhoB^+^ strain to the specific activity of β-galactosidase of the corresponding PhoB^−^ strain.
